# Metagenomic analysis of fecal and tissue samples from 18 endemic bat species in Switzerland revealed a diverse virus composition including potentially zoonotic viruses

**DOI:** 10.1371/journal.pone.0252534

**Published:** 2021-06-16

**Authors:** Isabelle Hardmeier, Nadja Aeberhard, Weihong Qi, Katja Schoenbaechler, Hubert Kraettli, Jean-Michel Hatt, Cornel Fraefel, Jakub Kubacki

**Affiliations:** 1 Institute of Virology, University of Zurich, Zurich, Switzerland; 2 Functional Genomics Center Zurich, Zurich, Switzerland; 3 Bat Foundation Switzerland, Zurich, Switzerland; 4 Clinic for Zoo Animals, Exotic Pets and Wildlife, University of Zurich, Zurich, Switzerland; Ohio State University College of Medicine, UNITED STATES

## Abstract

Many recent disease outbreaks in humans had a zoonotic virus etiology. Bats in particular have been recognized as reservoirs to a large variety of viruses with the potential to cross-species transmission. In order to assess the risk of bats in Switzerland for such transmissions, we determined the virome of tissue and fecal samples of 14 native and 4 migrating bat species. In total, sequences belonging to 39 different virus families, 16 of which are known to infect vertebrates, were detected. Contigs of coronaviruses, adenoviruses, hepeviruses, rotaviruses A and H, and parvoviruses with potential zoonotic risk were characterized in more detail. Most interestingly, in a ground stool sample of a *Vespertilio murinus* colony an almost complete genome of a Middle East respiratory syndrome-related coronavirus (MERS-CoV) was detected by Next generation sequencing and confirmed by PCR. In conclusion, bats in Switzerland naturally harbour many different viruses. Metagenomic analyses of non-invasive samples like ground stool may support effective surveillance and early detection of viral zoonoses.

## Introduction

Bats belong to the order Chiroptera, a group of mammals with 21 families and more than 1’300 species of which approximately 1’236 are classified by the International Union for Conservation of Nature (IUCN) [1–4]. Bats are one of the most diverse and abundant groups of animals, living all over the world except the Arctic and Antarctic [5, 6]. Nevertheless, nearly a third of all bat species are endangered [1, 7–9]. Bat species have adapted to various food sources such as arthropods, fruit, nectar, pollen, small mammals, fish, frogs, and blood, and they found a niche to navigate and hunt in darkness with the ability of echolocation and flight [10]. Bats play a key role in the ecosystem by pollinating flowers and dispersing seeds [10–12]. Five different families of insectivorous microbats are endemic in Europe, including *Vespertilionidae*, *Molossidae*, *Rhinolophidae*, *Pteropodidae* and *Emballonuridae*. Of the approx. 53 bat species identified in Europe, 30 are also endemic in Switzerland [5–7, 13, 14].

Bats have unique biological, physiological, and immunological characteristics that seem to make them ideal candidates to host and spread viruses. First of all, they are the only mammals capable of powered flight; some species migrate several hundred miles for winter hibernation enabling viral spread over long distances [15–17]. Also some species native to Switzerland, including *Nyctalus noctula*, *N*. *leisleri* and *N*. *lasiopterus*, *Vespertilio murinus* and *Pipistrellus nathusii*, are known to fly long distances [7]. Second, the longevity, i.e. up to 41 years (*Myotis brandtii*), facilitates long virus persistence [18–21]. Third, bats roost in colonies with a size of up to a million animals per colony which supports virus transmission between individuals [16]. Moreover, as bats are phylogenetically the oldest mammals on earth, their immune system was able to adapt and cope with viral infections over a long time of co-evolution. Bats seem to control viral replication more effectively than other mammals [22, 23]. It has been shown that due to the development of flight, the immune system adapted to the high metabolic rates i.e., increased metabolism and high body temperature during flight, through genetic changes. Flight would therefore lead to a daily activation of the immune system and could explain why bats can host viruses with no signs of illness [16, 22, 24, 25]. The large number of different bat species may also contribute at least in part to the large number of different bat-associated zoonotic viruses [26, 27].

Bats are not only ideal hosts and reservoirs for many different viruses, but in addition several factors also support the efficient transmission of viruses. These include intrinsic factors such as body condition, sex, or social and reproductive status as well as extrinsic factors influencing the habitat of the bats such as drought, cave destruction, and bush fires [16, 18, 28–30]. Stress (e.g., starving, mating, breeding, fighting, high population density) amplifies virus transmission from bat to bat even further as it downregulates the immune system and makes the animals more susceptible to viral infections, which has a direct impact on the epidemiology of viral diseases [16, 18, 28–30]. Habitat loss and lack of food often forces bats to move closer to domestic areas, thereby increasing the chance of virus transmission to other species including humans and farm animals [18].

Many different viruses have been detected in bats using a variety of assays, including serological tests, polymerase chain reaction (PCR) and virus isolation [16, 31–41]. Among those, countless viruses from insects, plants, fungi, and bacteria mainly reflect the dietary habits of the bats [42–44]. But bats are also the natural hosts for many different vertebrate viruses including emerging zoonotic viruses such as Ebola virus, Marburg hemorrhagic fever filoviruses, rabies virus, severe acute respiratory syndrome coronavirus (SARS-CoV), Middle-East respiratory syndrome coronavirus (MERS-CoV), Nipah (NiV) and Hendra virus (HeV) [14, 18, 24, 32, 38, 45–48]. Up to date, 20 out of 29 virus families associated with bats were detected in Europe, and the different viruses were compiled in the database DBatVir [49, 50]. Viruses from at least 12 families found in Europe have zoonotic potential, but direct bat-to-human transmission has been reported only for lyssaviruses and so far with a low prevalence [14, 32]. For transmission of bat borne viruses to humans often an intermediate host is needed [47, 51, 52]. For example in the coronavirus outbreaks SARS-CoV-1 and MERS-CoV, the viruses originated from bats but were transmitted to humans through an intermediate host i.e. civets and dromedary, respectively [46, 48, 53, 54]. The pandemic caused by SARS-CoV-2 presumably originated from a bat coronavirus with a 96% nucleotide similarity to the SARS-CoV-2 detected in a bat from the province Yunann (SARSr-CoV; RaTG13) [55, 56]. Coronaviruses circulate widely in wildlife and the bat-to-human transmission and the role of the intermediate host in transmission is still unclear [57]. However, some reports suggest that the Malayan pangolin (*Manis javanica*) may serve as an intermediate host of SARS-CoV-2 [55, 56, 58, 59].

While the virus diversity of bats has been determined in several countries including China, Japan, Myanmar, the USA and France [5, 43, 60–63], no such data is available from Switzerland. Therefore, in a collaboration with the foundation for bat conservation Switzerland and local care takers of bat colonies, more than 7’000 bats were sampled for the metagenomic study presented here.

## Material and methods

### Animals and sample collection

Samples were collected between 2015 and 2020 from bats presented at care centers in the cantons Zurich, Basel, Berne, Grisons, Jura, Neuchatel, Lucerne, Schaffhausen, and St. Gallen and from selected bat colonies in the cantons Aargau, Jura, Lucerne, Obwalden, Schaffhausen, Schwyz, Solothurn, St. Gallen, and Zurich (S1 Fig). The geographic coordinates of bat colonies can be provided by the Swiss Bat Conservation Foundation and bat care centers upon formal request. On living bats, only non-invasive sampling procedures (feces collection from the floor) were performed. Fecal samples of individual bats were collected at the bat care center located at the Zoo Zurich, where injured animals or animals that were disturbed during hibernation are housed in boxes. During routine cleaning of the boxes, fresh fecal samples were collected and stored in 2 ml tubes. Fecal samples from bat colonies were collected from the floor and stored in 50 ml tubes during routine inspection by authorized persons when the animals were present. Organs were collected from bats which were found dead or brought to the bat care centers and due to injuries had to be euthanized by veterinarians. For this, the animals were anesthetized with 20 mg/kg Alfaxalone (Alfaxan) s.c. in the neck area and, after 10min, euthanized with Pentobarbital (Esconarkon; 0.1–0.2 ml / animal) i.p. From necropsy, brain, heart, lung, intestine, and spleen combined with liver were collected and stored separately in 2-ml tubes. The samples of individual animals were pooled if they matched concerning three specific criteria: (i) the species, (ii) the sample type and (iii) the location (canton). All other samples from individual animals were prepared separately. Ground stool samples of colonies were not pooled. All samples were stored at -20°C. Table 1 shows an overview of the samples analyzed in this study; for more detail see S1–S3 Tables. Ethical approval was not required because only non-invasive sampling procedures on living bat were performed, and corpses were from natural deaths or from bats that had to be euthanized due to injuries. The euthanasia, thus, was not part of this study.

**Table 1:** Place of collection, bat species, numbers of bats, and samples collected in this study.

		Numbers of animals	
Canton	Bat species	Feces	Organs
Aargau	*Myotis daubentonii*	200^a^	
	*Myotis myotis*	2’454^a^	11
	*Myotis mystacinus*	50^a^	2
	*Myotis nattereri*	50^a^	
	*Nyctalus noctula*	200^a^	
	*Pipistrellus nathusii*	4	
	*Pipistrellus kuhlii*	1	
	*Pipistrellus pipistrellus*		1
	*Pipistrellus sp*.	1	
	*Rhinolophus ferrumequinum*	5^a^	
	*Vespertilio murinus*	200^a^	
Basel	*Pipistrellus kuhlii*		7
	*Pipistrellus nathusii*		10
	*Pipistrellus pipistrellus*	1	16
Berne	*Plecotus auritus*		1
Grisons	*Eptesicus nilssonii*		3
	*Myotis nattereri*		1
	*Nyctalus leisleri*		2
	*Pipistrellus nathusii*		1
	*Pipistrellus pipistrellus*		2
	*Plecotus auritus*		2
	*Plectous macrobullaris*		1
	*Rhinolophus ferrumequinum*	186^a^	
	*Rhinolophus hipposideros*	12^a^	
Jura	*Myotis myotis*	600^a^	
	*Pipistrellus pipistrellus*		1
Lucerne	*Myotis daubentonii*	1	
	*Myotis myotis*	778^a^	1
		2	
	*Pipistrellus nathusii*	1	
	*Pipistrellus pygmaeus*	1	1
Neuchâtel	*Myotis myotis*	2	
	*Pipistrellus nathusii*	2	
	*Pipistrellus pipistrellus*	2	1
Obwalden	*Myotis myotis*	250^a^	
Schaffhausen	*Plecotus austriacus*	1	
	*Myotis myotis*	370^a^	
Schwyz	*Myotis myotis*	212^a^	
Solothurn	*Myotis myotis*	208^a^	
St. Gallen	*Myotis myotis*	310^a^	
	*Plecotus auritus*		1
	*Pipistrellus kuhlii*		1
Uri	*Myotis myotis*	300^a^	
Zurich	*Myotis daubentonii*	4	3
	*Myotis myotis*	568^a^	
		1	
	*Myotis mystacinus*	1	1
	*Nyctalus noctula*	72	
	*Pipistrellus kuhlii*	8	11
	*Pipistrellus nathusii*	7	5
	*Pipistrellus pipistrellus*	13	14
	*Pipistrellus sp*	7	4
	*Plecotus auritus*	4	2
	*Vespertilio murinus*	1	2
Liechtenstein	*Myotis myotis*	93^a^	
Total		7’183	108

^a^ ground stool sample of colony.

### Enrichment of viral particles and nucleic acid isolation

#### Homogenization and filtration

To homogenize fecal samples from individual animals or small groups, 360 μl of phosphate buffered saline (PBS) was added to 30 mg of feces. To homogenize organs, 300–500 μl of PBS was added to 30 mg of tissue from up to 10 individual animals grouped according to species, geographical location, and organ type. Complete homogenization of tissue samples was achieved by adding a stainless-steel bead of 5 mm diameter (Qiagen, Hombrechtikon, Switzerland) into each tube. Then, samples were homogenized in the TissueLyser II (Qiagen, Hombrechtikon, Switzerland) at 20 Hz for 2 min, followed by 5 min of centrifugation at 16’060 x *g* (Biofuge Fresco, Heraeus Instruments, Hanau, Germany). The supernatants were collected with a 5-ml syringe (Injekt F, B. Braun, Sempach, Switzerland) and a 22 G needle (0.7 x 32 mm, AGANI NEEDLE, Terumo, Eschborn, Germany) and filtered through a 0.45 μm syringe filter (Puradisc 13 mm, Whatman GE Healthcare, Chicago, Illinois, USA) to remove bacterial and host cells. If clogging occurred, filtration was repeated. After the filtration step, pools of tissues from animals of the same species and geographical location were combined in 1.5-ml Eppendorf tubes. Ground stool samples from colonies were placed in a petri dish and cut into pieces with a scalpel blade (carbon steel sterile surgical blade#18, B. Braun, Sempach, Switzerland), and the sample material was divided evenly into two new 15-ml tubes. One aliquot was stored at -20°C as backup and the other used for homogenization. For this, 12 ml of PBS was added, the sample mixed with a vortex, and then centrifuged for 10 min at 21’890 x *g* (Hereus Multifuge 3 S-R, Thermo Fisher, Waltham, Massachusetts, USA). The supernatant was transferred into a 2-ml tube and centrifuged a second time for 8 min at 16’060 x *g*. Then, the supernatant was taken out with a 5-ml syringe (Injekt F, B. Braun, Sempach, Switzerland) and 22 G needle (0.7 x 32 mm, AGANI NEEDLE, Terumo, Eschborn, Germany) and filtered first through a 0.8 μm syringe filter (13 mm Supormembrane, Pall Corporation, New York, USA) and then through a 0.45 μm syringe filter (Puradisc 13 mm, Whatman, GE Healthcare, Chicago, Illinois, USA) into 1.5-ml Eppendorf tubes [64].

#### Nuclease treatment

To remove nucleic acids not protected by a virus coat, 134 μl of each filtered homogenate from the step above was treated with 1 μl of micrococcal nuclease (2 x 10^6^ gel U/ml; New England Biolabs, Ipswich, Massachusetts, USA), 14 μl of 10 X micrococcal nuclease buffer and 1 μl of Ribonuclease A (30mg/ml; Sigma-Aldrich, St. Louis, Missouri, USA). Then, the samples were incubated for 15 min at 45°C and for 1 h at 37°C [64].

#### Nucleic acid isolation

Viral RNA and DNA was prepared by using the QIAmp Viral RNA Mini kit (Qiagen, Hombrechtikon, Switzerland) as described by the manufacturer with modifications: the RNA carrier was omitted and, as a first step, 594 μl of AVL buffer was mixed with 6 μl of 1% β-mercaptoethanol (Bio-rad, Hercules, California, USA). The nucleic acid was eluted with 20 μl of RNase free water and 20 μl of Tris-EDTA buffer (TE) [64, 65].

### Reverse transcription and second strand synthesis

For cDNA synthesis, 2.5 μM of a random primer with a known 20-nt tag sequence (SISPA-N, 5’-GCT GGA GCT CTG CAG TCA TCN NNN NN-3’) was added to the nucleic acid samples prepared in the step above, incubated at 97°C for 3 min, and cooled on ice. Then 20 μl of cDNA-mix (RevertAid First Strand H minus cDNA Synthesis kit; Thermo Fisher, Waltham, Massachusetts, USA), consisting of 10 μl of 5 X reaction buffer, 5 μl of 10 mM dNTP mix, 2.5 μl of 20U/ μl Ribolock RNase inhibitor, and 2.5 μl of 200U/ μl RevertAid H minus RT, was added to each sample, and the suspension was incubated for 10 min at 25°C, 90 min at 45°C, and 5 min at 70°C. Finally, 1 μl of RNase H (New England Biolabs, Ipswich, Massachusetts, USA) was added to degrade residual RNA, and the sample was incubated for 20 min at 37°C. For second strand synthesis, 45.5 μl of cDNA, 0.6 μl of 10 X Klenow buffer (Thermo Fisher, Waltham, Massachusetts, USA), 0.4 μl of 100 μM SISPA-N primer, and 1 μl of 10 mM dNTP were mixed, denatured for 1 min at 95°C and cooled on ice. Then, 2.5 μl of Klenow Fragment 3’→5’ exo- (Thermo Fisher, Waltham, Massachusetts, USA) was added, and the mixture was incubated for 15 min at 25°C and for 1 h at 37°C and subsequently denatured for 1 min at 95°C. Following cooling on ice, another 1.25 μl of Klenow Fragment 3’→5’ exo- was added and the reaction was allowed to proceed for 15 min at 25°C and 1 h at 37°C. Samples were purified using the PureLink® PCR Micro kit (Invitrogen, Thermo Fisher, Waltham, Massachusetts, USA) according to the manufacturer’s protocol. Finally, dsDNA was eluted with 12 μl of buffer E1 (Invitrogen, Thermo Fisher, Waltham, Massachusetts, USA) [64].

### Random amplification

Before library preparation, dsDNA was amplified using a sequence-independent single primer (SISPA primer, 5’-GTT GGA GCT CTG CAG TCA TC-3’) and HotStarTaq DNA polymerase (5U/ μl; Qiagen, Hombrechtikon, Switzerland) as described previously [66]. Then, the samples were purified using the QIAquick PCR Purification kit (Qiagen, Hombrechtikon, Switzerland) according to manufacturer’s protocol and eluted with 30 μl of elution buffer (EB). The end concentrations of dsDNA in the samples were measured using a Qubit™ 2.0 Fluorometer (Invitrogen, Carlsbad, California, USA) [64].

### Library preparation

For fragmentation of the dsDNA and ligation of specific adaptors to the DNA fragments, the samples were diluted to a final concentration of 3 ng of DNA in 50 μl of EB buffer (or 1 ng per 50 μl for samples with low initial concentrations). The diluted samples were transferred into microTUBES (Covaris, Massachusetts, USA) and sonicated using an S220 Ultrasonicator System (Covaris, Massachusetts, USA) to obtain 500 bp fragments for paired end NGS runs, or 300 bp fragments for single end NGS runs, according to the manufacturer’s manual. For library preparation, the NEBNext Ultra II DNA Library Prep kit for Illumina (New England Biolabs, Ipswich, Massachusetts, USA) was employed according to the manufacturer’s manual. AMPure XP Beads (Beckman Coulter, Brea, California, USA) were used to clean up the adaptor-ligated DNA, and the NEBNext Multiplex Oligos (96 Unique Dual Index Primer Pairs; New England Biolabs, Ipswich, Massachusetts, USA) were used for the barcoding of the samples.

### Quality control and sequencing

The Agilent 2200 TapeStation (Agilent Technologies, Santa Clara, California, USA) was used with a D1000 HS ScreenTape (Agilent Technologies, Santa Clara, California, USA) to measure the size distribution and molarity of each sample. Sample denaturation, dilution, and sequencing were performed at the Functional Genomics Center Zurich (FGCZ) on either the NextSeq 500 system using the high-output flow cell with a paired-end NGS run and 2 x 150 nucleotide read length or the Illumina NovaSeq 6000 system using a single end NGS run with 100 nucleotide read length according to the protocols provided by the manufacturer (Illumina, San Diego, California, USA). The PhiX Control v3 Library as run quality control according to the manual (Illumina, San Diego, California, USA).

### Electron microscopy

For electron microscopy, selected fecal and tissue samples were subtilized in 15-ml tubes with 5 to 6 ml of PBS. Then, the same volume of 1-butanol was added prior to vortexing for 3 min and centrifugation for 30 min at 500–700 x *g* (Heraeus™ Multifuge™ X3, Thermo Fisher, Waltham, Massachusetts, USA). After incubation at 4°C for 5 h, the aqueous phase was transferred into a new 15-ml tube and topped up to 5 ml with PBS if the volume was smaller. Then, 5 ml (minimal ratio 1:1) of 1-butanol was added, and the mixture was vortexed for 3 min, centrifugated for 30 min at 500–700 x *g* (Heraeus™ Multifuge™ X3, Thermo Fisher, Waltham, Massachusetts, USA), and incubated for 5 h at 4°C. Again, the aqueous phase was transferred into a new 15-ml tube for mid-speed centrifugation, topped up with PBS to a final volume of 10 ml, and centrifugated for 20 min at 10’000 x *g* (Sorvall RC 5C PLUS using the HB-4 Rotor, Marshall Scientific, Hampton, New York, USA). The supernatant was transferred into an ultra-clear tube (14x95 mm, Beckmann Coulter, Brea, California, USA) and centrifugated for 2 h at 20’000 rpm and 4°C (60’000 x *g* Sorvall WX 100 ultra-series centrifuge using the SW40 Rotor, Thermo Fisher, Waltham, Massachusetts, USA). Then, the supernatant was discarded, and the pellet containing the viral particles was resuspended in 20 μl of PBS. For negative staining, a parafilm strip (Bemis Company, Inc., Neenah, Wisconsin, USA) was placed onto a smooth surface. Then, 10 μl of the resuspended virus particles, a drop of double distilled water (ddH_2_O filtered with 0.22 μm pore size), and a drop of 2% phosphotungstic acid (PTA; H3(P(W3O10)4)xH_2_O), pH 7.0 were pipetted side by side onto the parafilm. A grid (carbon coated parlodion film mounted on a 300 mesh/inch copper grid), which was glow discharged to make it hydrophilic, was placed with the carbon coated side onto the top of the sample drop. After 10 min, the grid was placed onto the top of the ddH_2_O drop for a few seconds, and finally, onto the PTA for 1 min. Then, PTA was gently removed using a filter paper, and the grid was placed into a transmission electron microscope (CM12, Philips, Eindhoven, Netherlands) equipped with a CCD camera (Ultrascan 1000, Gatan, Pleasanton, California, USA) for analysis at 100 kV.

### PCR

Selected viral contigs detected by NGS were confirmed as follows: RNA was prepared by adding 270 μl of PBS to 30 mg of fecal or tissue sample, followed by vortexing and centrifugation for 3 min at 16’060 x *g* (Biofuge Fresco, Heraeus Instruments, Hanau, Germany). Then, 150 μl of the supernatant was used for RNA extraction using the Qiagen RNA Mini kit (Qiagen, Hombrechtikon, Switzerland) according to the manufacturer’s manual. DNA from tissue and fecal samples was prepared using the Qiagen DNA Mini kit or the Qiagen DNA Stool kit, respectively, according to the manufacturer’s manual (Qiagen, Hombrechtikon, Switzerland).

#### Pan-corona OneStep RT-PCR

The Pan-corona OneStep RT-PCR was performed as described by Vijgen et al. [67]. Additionally, two set of primers covering different region of polymerase gene were designed based on obtained sequence. The following primers were used: set one: forward 5’- GTTGATGGCGTGCCATTTGT-3’, reverse 5’- ATTGAGGCAACCACCGTCAT-3’ and set two: forward 5’- GGTGATGCCACTACCGCATA-3’, reverse 5’- TGAGCAGAACTCGTGTGGAC-3’. As a positive control the porcine epidemic diarrhea virus isolate CV777 was used and as a negative control nuclease free water. The PCR product was analyzed by agarose gel electrophoresis. The expected band of approx. 251, 465 and 396 base pairs (bp), for pancorona, set 1 and set 2, respectively, were cut out with a scalpel blade, and the DNA was extracted using the Gel extraction kit (Qiagen, Hombrechtikon, Switzerland) according to the manufacturer’s manual and sequenced at Microsynth (Balgach, Switzerland).

#### Pan-adeno PCR

The Pan-adeno nested PCR was performed as described by Wellehan et al. [68] using 5 μl DNA, 0.4 μl of HotStarTaq Polymerase (5U/ μl, Qiagen, Hombrechtikon, Switzerland), 0.5 μl dNTP mix (10 mM), 2.5 μl of PCR-Buffer (10X), 0.25 μl each of Panadeno outer forward and reverse primer (100 μM) and 16.1 μl nuclease free water. For the second amplification, 2 μl of the reaction mixture from the first amplification were mixed with 0.4 μl of HotStarTaq Polymerase (5U/ μl, Qiagen, Hombrechtikon, Switzerland), 0.5 μl dNTP mix (10 mM), 2.5 μl of PCR-Buffer (10X), 0.25 μl each of Panadeno inner forward and reverse primer (100 μM) and 19.1 μl nuclease free water. As a positive control the canine adenovirus 1 was used and as a negative control nuclease free water. The PCR product was then analyzed by agarose gel electrophoresis. The expected band of approx. 318–324 bp was cut out with a scalpel blade, and the DNA was extracted and sequenced as described above.

#### Rotavirus H NSP5 RT-PCR

To amplify the NSP5 segment of Rotavirus H (RVH), specific primers were designed using Primer3Plus [69] with manual inspection in Clonemanager 9 (Sci-Ed Software, Cary, North Carolina, USA). As a negative control nuclease free water was used. The following primers were used: forward 5’-GGAACTAAAAACTTCAATCGTTGCTG-3’ and reverse 5’-GTTTTTATTGATGACCTCAGGGGC-3’. Before amplification, an initial denaturation step with 10 μl of extracted RNA for 5 min at 97°C was performed. To the denatured RNA 40 μl of the PCR mix containing 10 μl of One Step RT-PCR Buffer (5X; Qiagen, Hombrechtikon Switzerland), 1.6 μl forward Primer (100 μM) and 1.6 μl reverse Primer (100 μM), 2 μl dNTP Mix (10 mM each), 2.0 μl One Step Enzyme Mix (Qiagen Hombrechtikon, Switzerland), 22.6 μl nuclease free water, and 0.2 μl RNasin (Promega, Madison, Wisconsin, USA) was added. The PCR conditions were as follows: 30 min at 50°C, 15 min at 95°C, 40 cycles of 50 sec at 94°C, 50 sec at 55°C and 60 sec at 72°C, a final extension step for 10 min at 72°C. The PCR product was analyzed by agarose gel electrophoresis. The expected band of approx. 666 bp was cut out with a scalpel blade, and the DNA was extracted and sequenced as described above.

### Data analysis

Sequencing data was used in reference guided analysis and de novo assembly pipelines as described previously [65]. The PCR primers, sequencing adaptors, and low-quality ends in raw reads were trimmed using Trimmomatic (version 0.36) and cutadapt (version 2.9). Quality controlled reads were aligned using Bowtie2 (version 2.4.1) to the human genome to remove contamination introduced during sample preparation. This was followed by the assembly of un-mapped reads in a reference guided analysis to detect even viruses with low numbers of reads. Reads were first aligned to an inhouse database containing 37’400 full length viral genomes downloaded from the NCBI database and bat-associated viruses from DBatVir database [49] using Bowtie2 (parameters: -a—very-sensitive—no mixed—no discordant -X 1000), and mapped reads and mapped bases per viral genome were calculated using bedtools (version 2.29.2).The same inhouse database was used subsequently to align reads in the metagenomic pipeline of the SeqMan NGen v17 (DNAStar, Lasergene, Madison, Wisconsin, USA) in order to visualize and confirm assembled reads. Finally, to build up contigs in the de-novo analysis, quality-controlled raw reads were assembled using megahit (version 1.1.3) with multiple k-mers [70]. To annotate contigs taxonomically, assembled contigs were compared against the NCBI none-redundant nucleotide nt database [71] using default parameter settings of BLASTN (version 2.6.0+) [72] and taxonomically annotated. Hits were sorted by bit scores, and hits with e value ≤ 1 × 10−5 and bit score ≥ 100 were used for contig taxonomy annotation. The naïve best-hit-method was then used to obtain the specific taxa assignment per contig after manual inspection of the alignments. The resulting viral contigs were further investigated and aligned using MEGA X and Clone manager ver. 9 (Sci-Ed). Phylogenetic trees were constructed in Mega X using the Maximum Likelihood method with 1’000 bootstrap value and a cut-off of 70% [73].

### Accession numbers

The nucleotide sequences of selected viruses identified in this study have been registered at the GenBank under the following accession numbers: MT815927-MT815982 and MT818221. All raw sequencing data generated during this study were uploaded to the Sequence Read Archive (SRA) under accession number PRJNA693645.

## Results

Fresh fecal samples of bats living in Switzerland (individual animals, colonies) were collected between 2018 and 2020, and tissue samples were taken from dead or diseased bats which had to be euthanized between 2015 and 2020 (Table 1 and S1 Fig). The samples were grouped into 174 sample pools (69 feces and 105 organ pools) according to sample type, bat species and collection location and then sequenced by NGS (Table 1, S1–S3 Tables). The data will be presented in the following order (i) a general overview of the virome of Swiss bats, (ii) the virome composition of different bat species, (iii) differences in the virome composition according to the sample types, (iv) an overview of viral genome abundance, and (v) a focus on selected virus families of vertebrates including *Coronaviridae*, *Adenoviridae*, *Reoviridae*, *Parvoviridae*, *Circoviridae*, and *Hepeviridae*.

### Overview of the virome of Swiss bats

**Fig 1:**
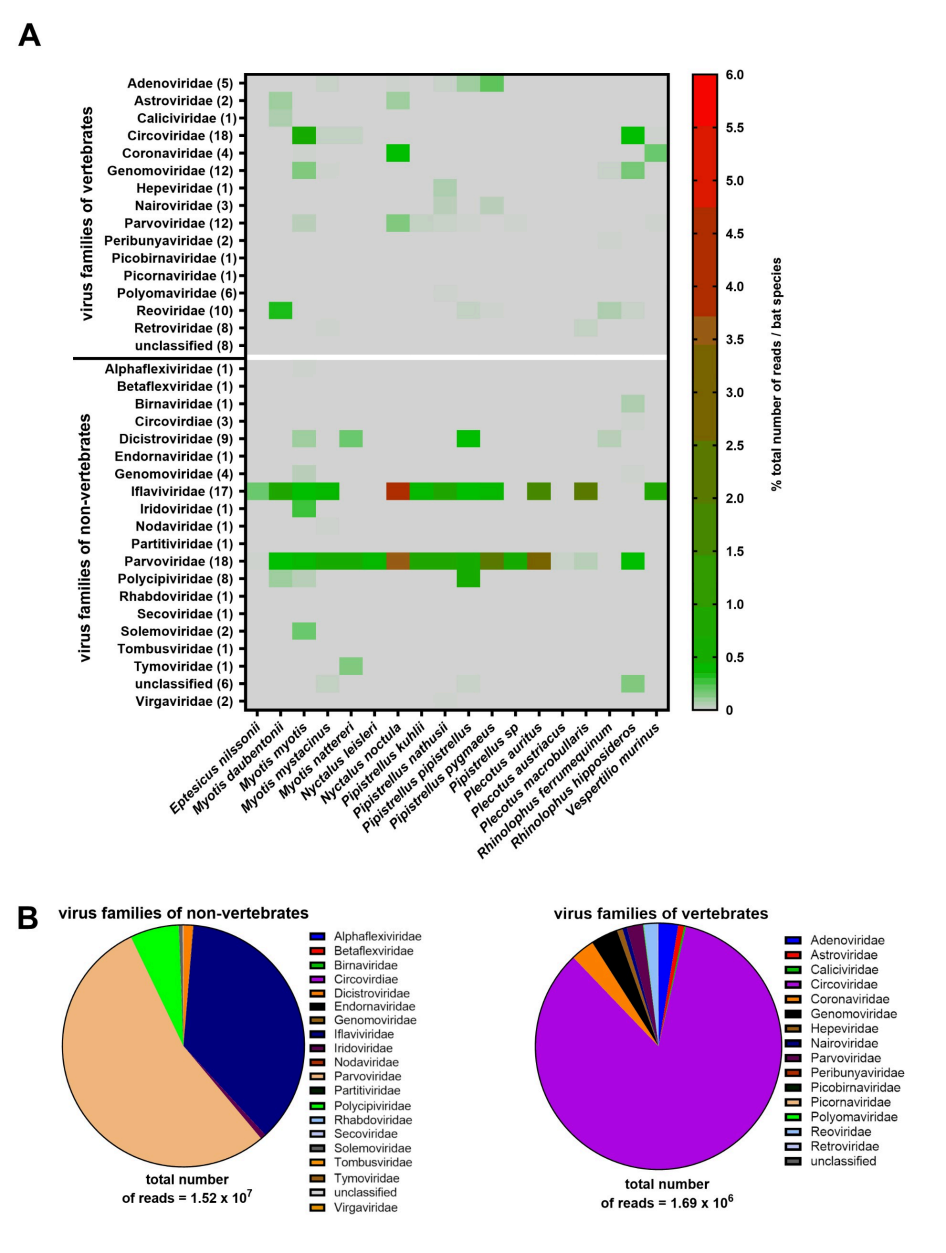
Overview of the virome of Swiss bats. **A.** Heatmap of viral reads from 16 virus families of vertebrates and 23 virus families of non-vertebrates and their distribution among all 18 bat species investigated shown as percentage of the total number of reads generated in each bat species. Number in parentheses indicate the numbers of different bat species in which sequences of a virus family have been detected. **B.** Read abundance of the different virus families.

The raw sequence data consisting of 1.28 x 10^9^ reads (0.5 x 10^6^ to 3.7 x 10^7^ from each pool) was analyzed using two different pipelines: (i) de novo assembly and (ii) reference-based assembly. The de novo assembly generated a total of 7’477 contigs matching to viral genomes. Using an inhouse data base, 1.68 x 10^7^ of the 1.28 x 10^9^ sequenced reads were assembled to virus genomes. Of these, 1.69 x 10^6^ (10%) reads matched to vertebrate viruses and 1.52 x 10^7^ (90%) to non-vertebrate viruses (Fig 1A and 1B). The generated sequencing reads were assembled to 39 virus families, i.e., 16 (41%) families of viruses from vertebrates and 23 (59%) families of viruses from non-vertebrates which include 11 (28.2%) families of insect-, 11 (28.2%) families of plant/fungal- and 1 (2.6%) family of environmental viruses.

#### Vertebrate viruses

**Fig 2:**
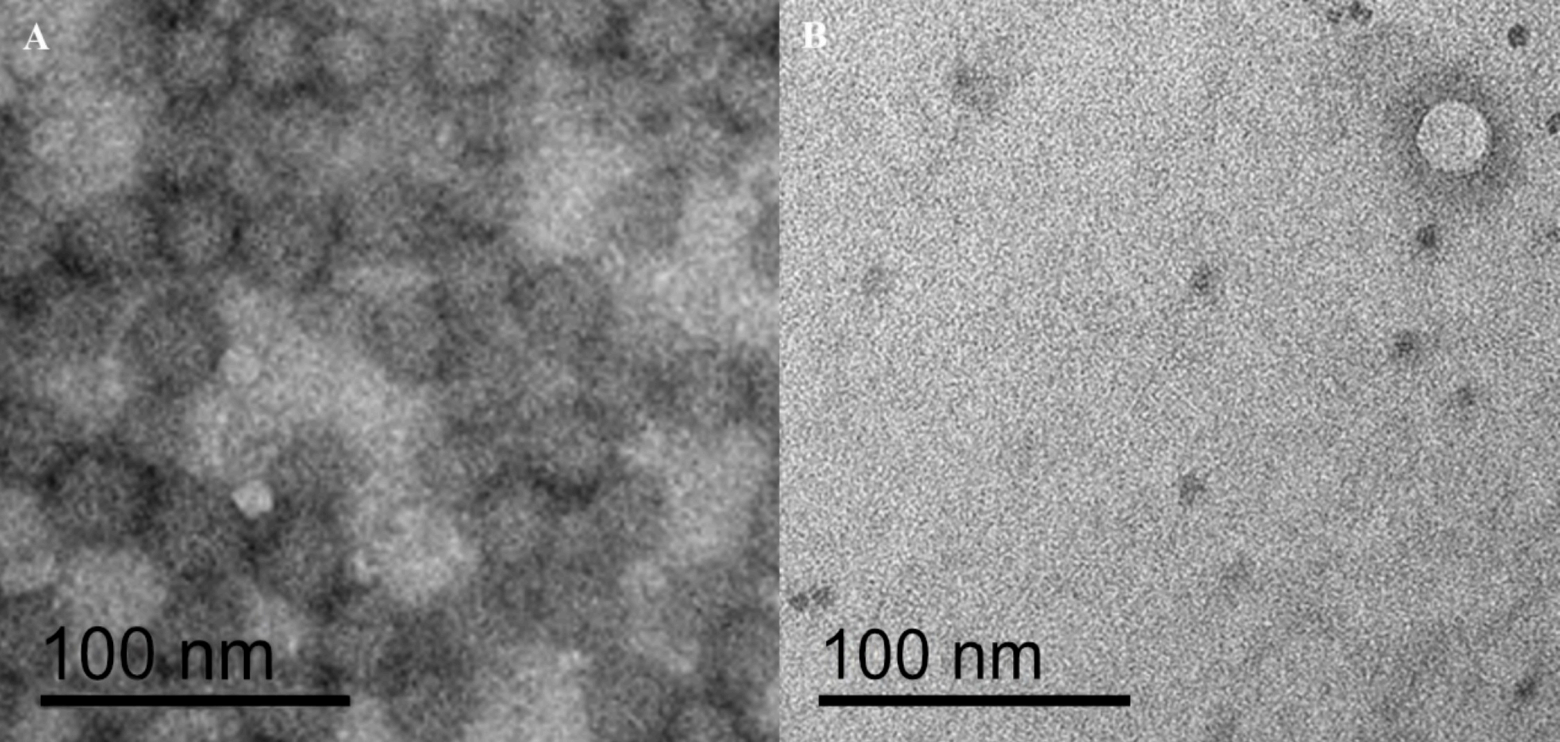
Electron microscopy of selected samples. **A.** Negative stain electron micrograph of a ground stool pool homogenate from a *Myotis myotis* colony. **B.** Negative stain electron micrograph of a pooled liver and spleen homogenate from *Pipistrellus nathusii*.

Reads assembled to the genomes of viruses of vertebrates included mainly the families of the *Circoviridae* (84.2%), *Genomoviridae* (3.6%), *Coronaviridae* (3.1%) and *Adenoviridae* (2.6%) (Fig 1A and 1B). Electron microscopy of a ground stool sample of a *Myotis myotis* colony with 1.3 x 10^6^ reads assembled to a circovirus sp. indeed revealed particles with a size (approx. 20nm) and shape (icosahedral/round) resembling that of circoviruses (Fig 2A) and an estimated particle concentration of > 10^12^ particles per ml (Fig 2A).

#### Insect viruses

Reads assembled to virus families infecting insects included mainly *Parvoviridae* (54.3%), *Iflaviridae* (37%) and *Polycipiviridae* (6.6%) (Fig 1A and 1B). More than 6 x 10^6^ reads were assembled to the subfamily *Densovirinae*, i.e., parus major densovirus, which was detected in 141 samples. A full-length genome of 5’164 nucleotides (nt) assembled from 6 x 10^5^ sequencing reads of an intestine pool of *Pipistrellus sp*. had a 97% nt similarity to a previously described parus major densovirus (GenBank acc. NC_031450). Interestingly, reads assembled to parus major densovirus have been detected also in several tissue pools including lung, liver/spleen, and intestine. Electron microscopy of a pooled liver/spleen homogenate from *Pipistrellus nathusii* tissue revealed particles with a size (approx. 22nm) and shape (icosahedral/round) resembling that of densovirus particles (Fig 2B). Additionally, several bee viruses were detected in ground stool samples of *Myotis myotis* colonies.

#### Plant/Fungal viruses

Reads assembled to genomes of viruses infecting plants or fungi included mainly the families of the *Solemoviridae* (88.1%), *Tymoviridae* (9%) and *Alphaflexiviridae* (1.8%) (Fig 1A and 1B).

### The virome composition of the different bat species

The sequences of two different virus families were detected in all 18 bat species, the *Circoviridae* and *Densovirinae*. The sequences of *Iflaviridae* were identified in 17, of *Genomoviridae* and vertebrate associated *Parvoviridae* in 12, and of *Reoviridae* in 10 bat species. *Pipistrellus pipistrellus* was the bat species with the largest variety of different virus families detected (11 virus families of vertebrates/11 virus families of non-vertebrates; 11/11), followed by *Myotis myotis* (8/10), *Pipistrellus nathusii* (9/7), *Myotis daubentonii* (8/4) and *Plecotus auritus* (7/5) (Fig 1A). Among the 18 species of bats, *Myotis myotis* represented the largest sample group with 87.4% of all sampled animals and was the species with the highest number of reads assembled to vertebrate viruses (0.5% of total generated sequencing reads), as well as the species with the largest number, 33, of different viruses detected (Fig 1A and S2 Fig). The second largest number of different viruses, 24, was detected in *Pipistrellus pipistrellus* (of which 12 were viruses of vertebrates and 12 of non-vertebrates), followed by *Pipistrellus nathusii* and *Myotis mystacinus* with 17 different viruses each. The lowest number of vertebrate viral reads were detected in *Plecotus austriacus* and of non-vertebrate viral reads in *Rhinolophus ferrumequinum* i.e., 1.45 x10^-5^ and 6.8 x 10^−3^ of total generated reads, respectively.

Five bat species in Switzerland are known to migrate i.e., *Nyctalus leisleri*, *Nyctalus lasiopteros*, *Nyctalus noctula*, *Pipistrellus nathusii* and *Vespertilio murinus*. Samples from all migrating species except *Nyctalus lasiopteros* were collected. Between migrating and non-migrating bats only minor differences in the virus genome diversity and the numbers of reads were observed (S2 Fig). The average numbers of different viruses detected in migrating bats was lower than in non-migrating bats. On the other hand, the average number of different viruses of vertebrates was higher in migrating than in non-migrating bats (Fig 1A and 1B, and S2 Fig). *Nyctalus noctula* was the species with highest percentage of non-vertebrate viral reads detected (7.27% of the total generated reads) (S2 Fig).

### Differences in the virome composition according to the sample types

In general, more viral reads were detected in fecal and intestine samples of individual animals than in tissue samples (brain, lung, and combined liver/spleen). The largest number of viral reads assembled to vertebrate virus families were detected in ground stool samples from colonies, whereas reads assembled to non-vertebrate viruses were most abundant in fecal samples of individual animals. Specifically, *Retroviridae* and *Polyomaviridae* sequences were detected mainly in tissue samples while *Nairoviridae* sequences were only detected in tissue pools and *Astroviridae*, *Caliciviridae* and *Coronaviridae* sequences were not detected in tissue samples. In the intestine pool and tissue pool, families of vertebrate viruses were the most abundant, 13 and 11, respectively. In the ground stool samples, families of non-vertebrate viruses were the most abundant, 14. All samples had in common that more reads were assembled to non-vertebrate than to vertebrate viruses (Table 2).

**Table 2:** Overview of reads assembled to different virus families categorized by sample type.

	Sample type					
Virus family	Feces (individual animals)	Ground stool (colonies)	Intestine pool	Tissue pool	
				Lung pool	Liver/Spleen pool	Brain pool
Vertebrate						
*Adenoviridae*	1.4 x 10^4^	0	2.5 x 10^4^	0	3.7 x 10^3^	0
*Astroviridae*	1.3 x 10^4^	0	3	0	0	0
*Caliciviridae*	2 x 10^3^	0	1.9 x 10^3^	0	0	0
*Circoviridae*	970	1.4 x 10^6^	1.1 x 10^3^	80	1.4 x 10^3^	351
*Coronaviridae*	3.6 x 10^4^	1.7 x 10^4^	377	0	0	0
*Genomoviridae*	207	6 x 10^4^	69	14	3	0
*Hepeviridae*	1.3 x 10^4^			0		
*Nairoviridae*	0	0	0	0	1 x 10^4^	0
*Parvoviridae*	2.2 x 10^3^	2.6 x 10^4^	4.1 x 10^3^	109	2.9 x 10^3^	0
*Peribunyaviridae*	0	353	0	0	0	0
*Picobirnaviridae*	0	0	99	0	0	0
*Picornaviridae*	0	0	2	0	0	0
*Polyomaviridae*	0	386	17	0	1 x 10^3^	0
*Reoviridae*	1.8 x 10^4^	2.5 x 10^3^	8.7 x 10^3^	2	271	0
*Retroviridae*	6	0	87	1.4 x 10^3^	487	233
Unclassified	66	620	47	0	0	3
Total	1 x 10^5^	1.5 x 10^6^	4.1 x 10^4^	1.6 x 10^3^	2 x 10^4^	587
Non-vertebrate						
Alphaflexiviridae	0	1.5 x 10^3^	0	0	0	0
Betaflexviridae	0	51	0	0	0	0
Birnaviridae	0	799	0	0	0	0
Circovirdiae	593	123	5	0	0	0
Dicistroviridae	0	4.4 x 10^4^	1.4 x 10^5^	81	299	0
Endornaviridae	0	0	23	0	0	0
Genomoviridae	21	1.6 x 10^4^	0	0	0	0
Iflaviviridae	4.8 x 10^6^	8.7 x 10^4^	6.4 x 10^5^	1.9 x 10^4^	2 x 10^4^	17
Iridoviridae	0	1.1 x 10^5^	0	0	0	0
Nodaviridae	0	330	0	0	0	0
Partitiviridae	1	0	0	0	0	0
Parvoviridae	5 x 10^6^	5.8 x 10^5^	2.4 x 10^6^	4 x 10^4^	1.3 x 10^5^	165
Polycipiviridae	83	2.6 x 10^4^	9.6 x 10^5^	31	1.5 x 10^3^	8
Rhabdoviridae	0	0	0	0	100	0
Secoviridae	0	0	0	13	0	0
Solemoviridae	0	7.6 x 10^4^	7	0	0	0
Tombusviridae	33	0	0	0	0	0
Tymoviridae	0	7.8 x 10^3^	0	0	0	0
unclassified	25	1.9 x 10^3^	6 x 10^3^	0	0	6
Virgaviridae	653	0	114	0	48	0
Total	9.8 x 10^6^	9.6 x 10^5^	4.2 x 10^6^	6 x 10^4^	1.5 x 10^5^	196
**Total vertebrate/non-vertebrate**	**9.9 x 10**^**6**^	**2.5 x 10**^**6**^	**4.2 x 10**^**6**^	**6.1 x 10**^**4**^	**1.7 x 10**^**5**^	**783**

The total numbers of reads assembled to each virus family was categorized according to the different sample types including fecal samples of individual animals or pools of fecal samples from individual animals, ground stool samples of colonies, intestine pools, and tissue pools.

### Abundance of the different genome classes

**Fig 3:**
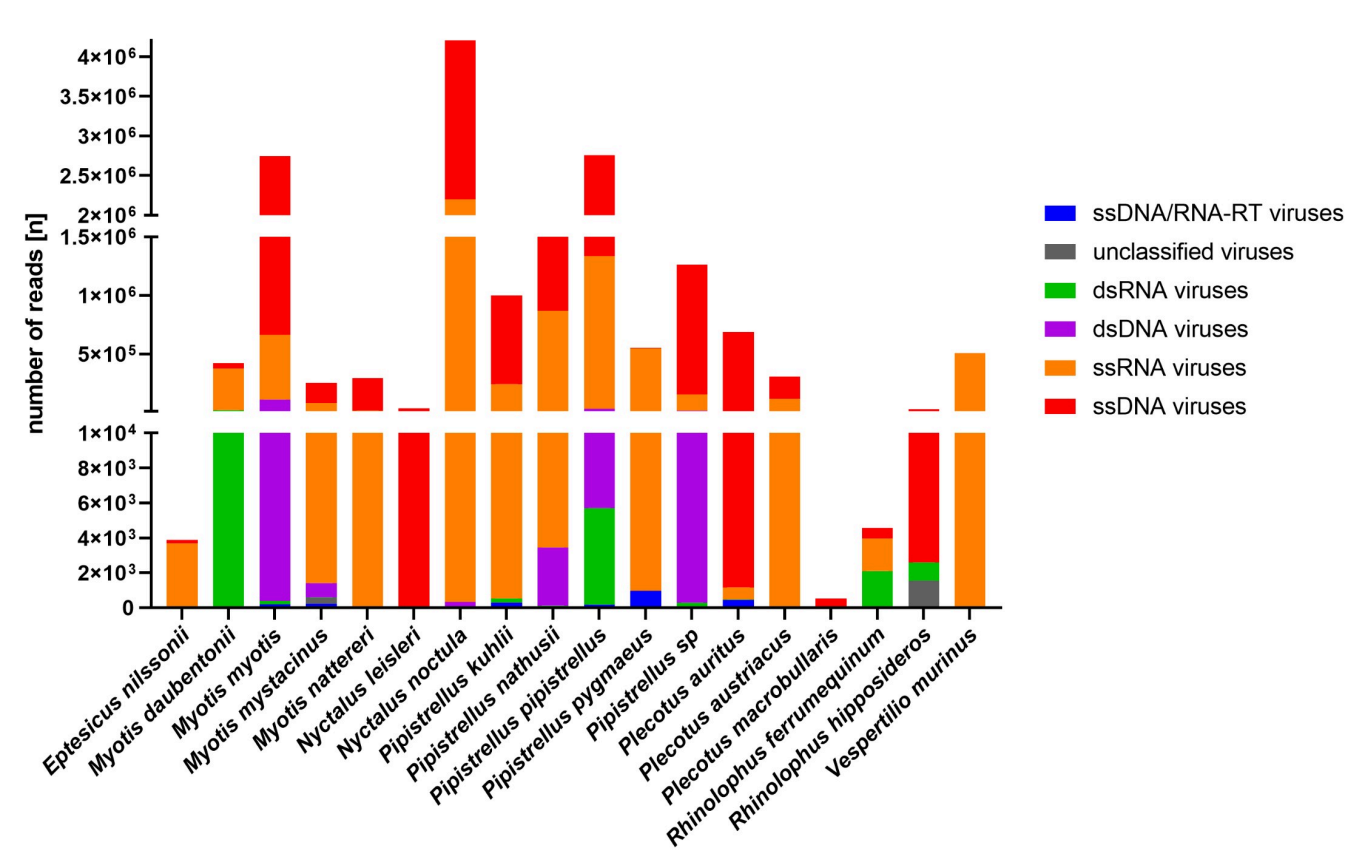
Distribution of viral reads according to genome classes. Because of the large differences in read numbers, the y-axis was divided into three parts to facilitate visualization of all virus genome classes in a single graph: part one from 0 to 1x 10^4^ reads, part two from 1x 10^4^ to 1.5x 10^6^ reads, and part three from 2x 10^6^ to >4.x 10^6^ reads.

Virus families from all genome classes were detected. Most virus families, 19, belong to the ssRNA viruses (19), followed by ssDNA viruses (8), and dsRNA viruses (5). Of the 19 families of ssRNA viruses, 7 infect vertebrates, 5 insects and 7 plants. Concerning the number of assembled reads the ssDNA viruses were the most abundant with 9.7 x 10^6^ assembled reads (57.7% of all assembled reads), followed by ssRNA viruses with 6.9 x 10^6^ assembled reads (41.2% of all assembled reads), and dsDNA viruses with 1.5 x 10^5^ assembled reads (0.92% of all assembled reads) (Fig 3). Sequences of ssDNA viruses were detected in all 18 bat species investigated, those of ssRNA viruses in 17, and those of dsRNA viruses in 11.

### Selected viruses of vertebrates

Viruses from 6 different virus families were studied in more detail i.e., coronaviruses (CoV), adenoviruses, rotaviruses A and H (RVA and RVH), parvoviruses (PV) and hepeviruses due to their potential zoonotic impact, and circoviruses because they were the most abundant among the viruses of vertebrates detected in the bat samples. Viruses mentioned above were detected in the single-end run on the Illumina NovaSeq 6000 system using a single end NGS run with 100 nucleotide read length.

#### Coronaviridae

*Coronaviridae* are enveloped viruses with a virion size of 120–160 nm for spherical morphology and of 75–90 nm x 170–200 nm for bacilliform morphology. The coronavirus genome consists of a single, linear segment of 26 to 32 kb of +RNA [74]. In our study reads assembled to *Coronaviridae* were identified in 17/174 (9.8%) sample pools. In ground stool pools of 13 *Myotis myotis* colonies and one *Vespertilio murinus* colony, in two fecal pools of *Nyctalus noctula*, and in one intestine pool of *Pipistrellus pipistrellus*, contigs of 614 to 20’189 nt in length were assembled that showed a high similarity (>87%) to different bat coronavirus genomes, mainly of the genus alphacoronavirus.

**Fig 4:**
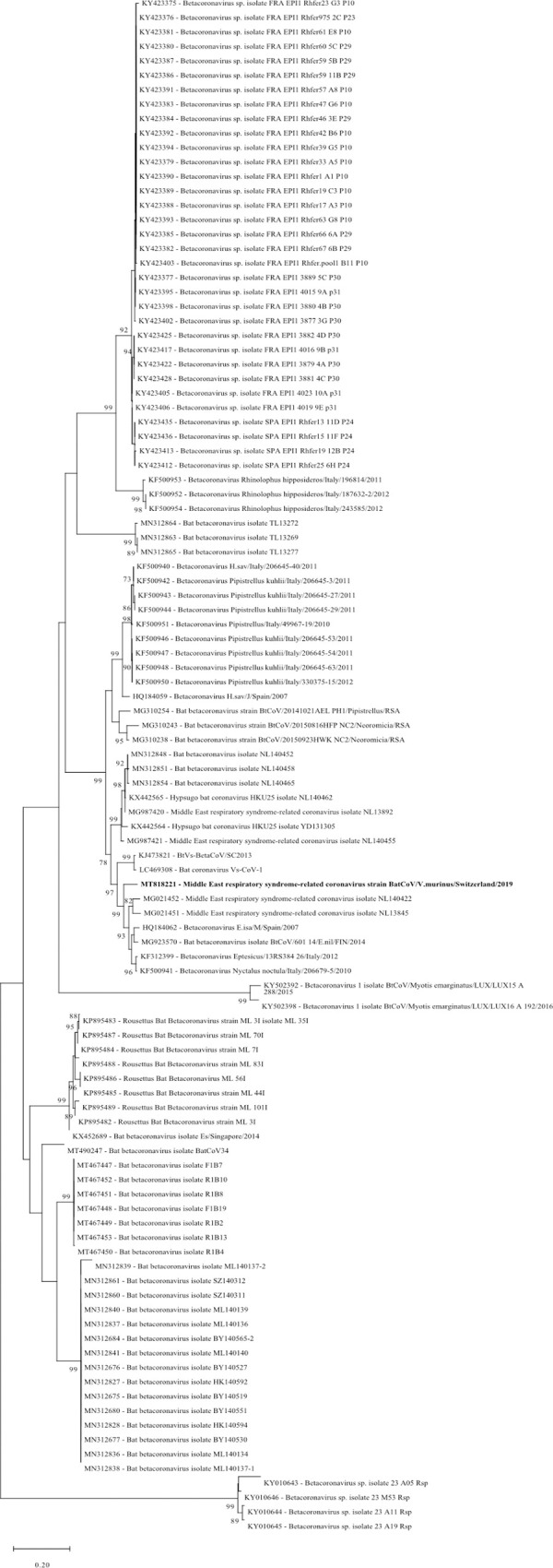
Phylogenetic analysis of the RNA dependent RNA polymerase gene of the bat betacoronaviruses. The sequence obtained in this study (GenBank acc. number MT818221) is shown in bold. Sequences from alphacoronaviruses are marked with a purple background and those of betacoronaviruses with a blue background. Sequences were aligned using Muscle. For phylogenetic analysis, the Maximum likelihood tree with 1’000 bootstraps was used. Only values ≥ 70% are displayed.

Additionally, in a ground stool sample of one *Vespertilio murinus* colony a contig of a bat coronavirus (GenBank acc. number MT818221) with a length of 20’189 nt was assembled that showed 86% nt similarity to a Middle East respiratory syndrome-related coronavirus (MERS-CoV) genome from China (GenBank acc. number MG021451) (Fig 4), a member of the genus betacoronavirus, subgenus Merbecovirus. The contig covered 3 ORF’s, including ORF1ab, ORF1a, and the ORF coding for the spike protein (S4 Table). Sanger sequencing of products obtained from pan-corona RT-PCR [67] and RT-PCR with primer sets 1 and 2 from the polymerase gene generated sequences of 158 nt, 443 nt and 392 nt, respectively, with 100% similarity to the contig generated by de novo assembly (GenBank acc. number MT818221).

#### Adenoviridae

**Fig 5:**
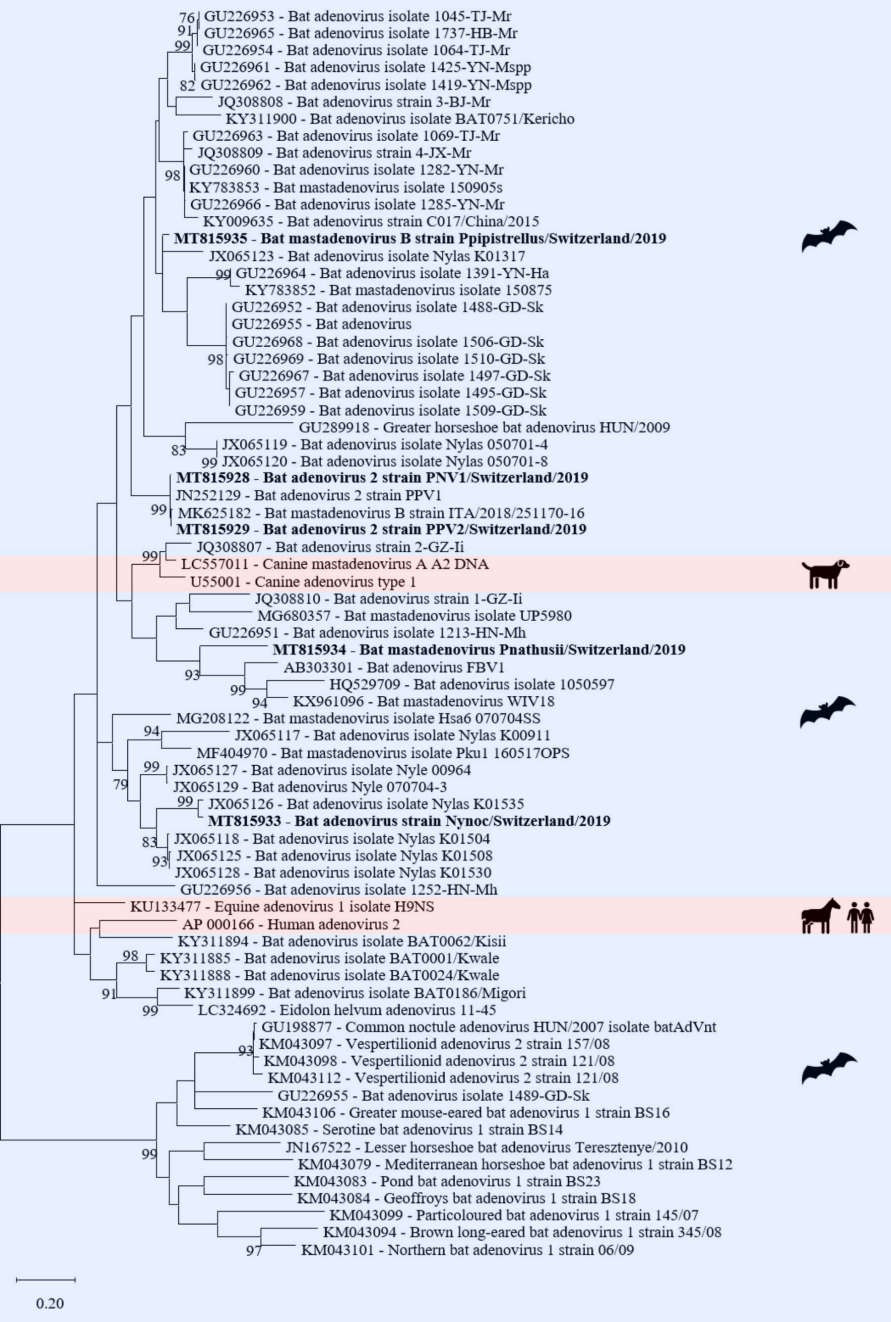
Phylogenetic analysis of the adenovirus DNA polymerase gene. The sequences obtained in this study (GenBank acc. numbers MT8159528-29, MT815933-35) are shown in bold. Sequences of non-bat associated viruses are marked with a red background and those of bat associated viruses with a blue background. The pictograms on the right side represent the species in which the virus was detected. Sequences were aligned using Muscle. For phylogenetic analysis, the Maximum likelihood tree with 1’000 bootstraps was used. Only values ≥ 70% are displayed.

*Adenoviridae* are non-enveloped viruses with an icosahedral capsid morphology and a virion size of 70–90 nm. The genome consists of a single segment of a linear ds-DNA of 26 to 48 kbp length [74]. Of the five genera of *Adenoviridae* only the genus mastadenovirus was detected in a fecal pool of *Pipistrellus nathusii*, a fecal pool of *Nyctalus noctula*, an intestine pool of *Pipistrellus pipistrellus*, an intestine pool of *Pipistrellus nathusii*, and a combined liver/spleen pool of *Pipistrellus pipistrellus*. Six assembled contigs (lengths between 2’184 and 14’493 nt) had 83 to 99% nt identity to the genome of a bat adenovirus 2 (GenBank acc. number JN252129) (S4 Table, Fig 5).

One contig with a length of 1’673 nt had 97% nt similarity to the DNA polymerase gene of a bat adenovirus (GenBank acc. number JX065126). Three assembled contigs had 71 to 83% nt similarity to the genomes of two bat mastadenoviruses (GenBank acc. number KX961096; MK625182) (S4 Table, Fig 5). Sanger sequencing of a pan-adeno PCR [68] product of the DNA polymerase gene generated sequences between 247–252 nt with a similarity between 79 and 100% to the contigs generated by de novo assembly of the sequencing reads.

#### Reoviridae

*Reoviridae* are non-enveloped viruses with an icosahedral capsid morphology and a virion size of 60–85 nm. The genome consists of 9 to 12 segments of dsRNA with a total genome size of 19 to 32 kbp. The *Reoviridae* includes viruses that infect vertebrates as well as viruses that infect non-vertebrate hosts [74]. In the bat samples RVA and RVH, which belong to the genus rotavirus, have been detected [75–77].

**Fig 6:**
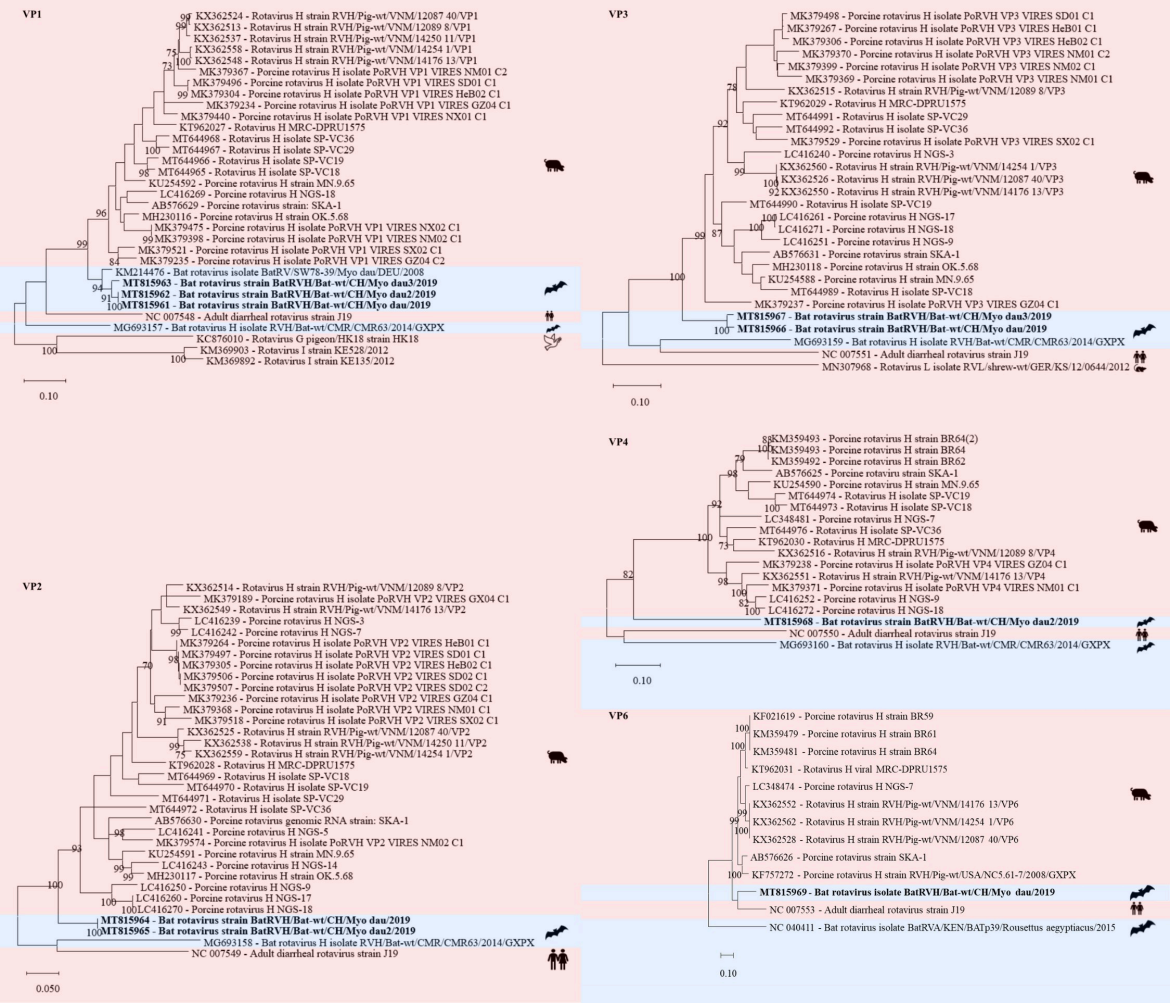
Phylogenetic analysis of specific regions of the rotavirus H genome. The sequences obtained in this study (GenBank acc. numbers MT815961-69) are shown in bold. Sequences of non-bat associated viruses are marked with a red background and those of bat associated viruses with a blue background. The pictograms on the right side represent the species in which the virus was detected. Sequences were aligned using Muscle. For phylogenetic analysis, the Maximum likelihood tree with 1’000 bootstraps was used. Only values ≥ 70% are displayed.

**Fig 7:**
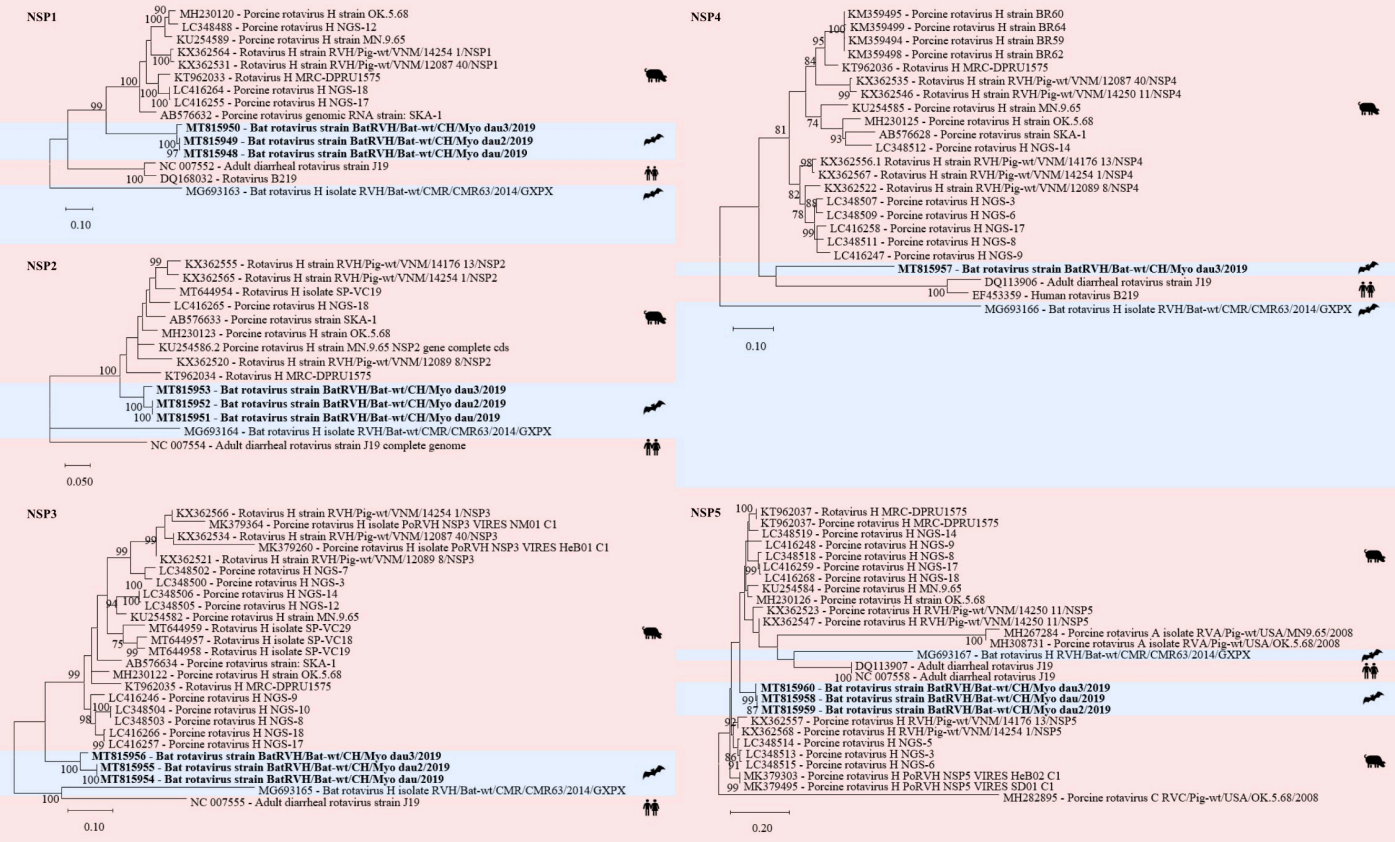
Phylogenetic analysis of specific regions of the rotavirus H genome. The sequences obtained in this study (GenBank acc. numbers MT815948-60) are shown in bold. Sequences of non-bat associated viruses are marked with a red background and those of bat associated viruses with a blue background. The pictograms on the right side represent the species in which the virus was detected. Sequences were aligned using Muscle. For phylogenetic analysis, the Maximum likelihood tree with 1’000 bootstraps was used. Only values ≥ 70% are displayed.

In three samples (two fecal pools, one intestine pool) of *Myotis daubentonii* 22 contigs were assembled to RVH each covering one to two segments, i.e. NS1 to NS5 and VP1 to VP4 and VP6 (S4 Table, Figs 6 and 7). Three contigs had a nt similarity between 92 and 93% to the NSP5 segment of a porcine RVH (GenBank acc. number KT962037) from South Africa (Fig 7). Sanger sequencing of a 666 bp RT-PCR product from the NSP5 segment generated sequences of 530–543 nt with 99 to 100% similarity to the contigs generated by de novo assembly of the sequencing reads.

**Fig 8:**
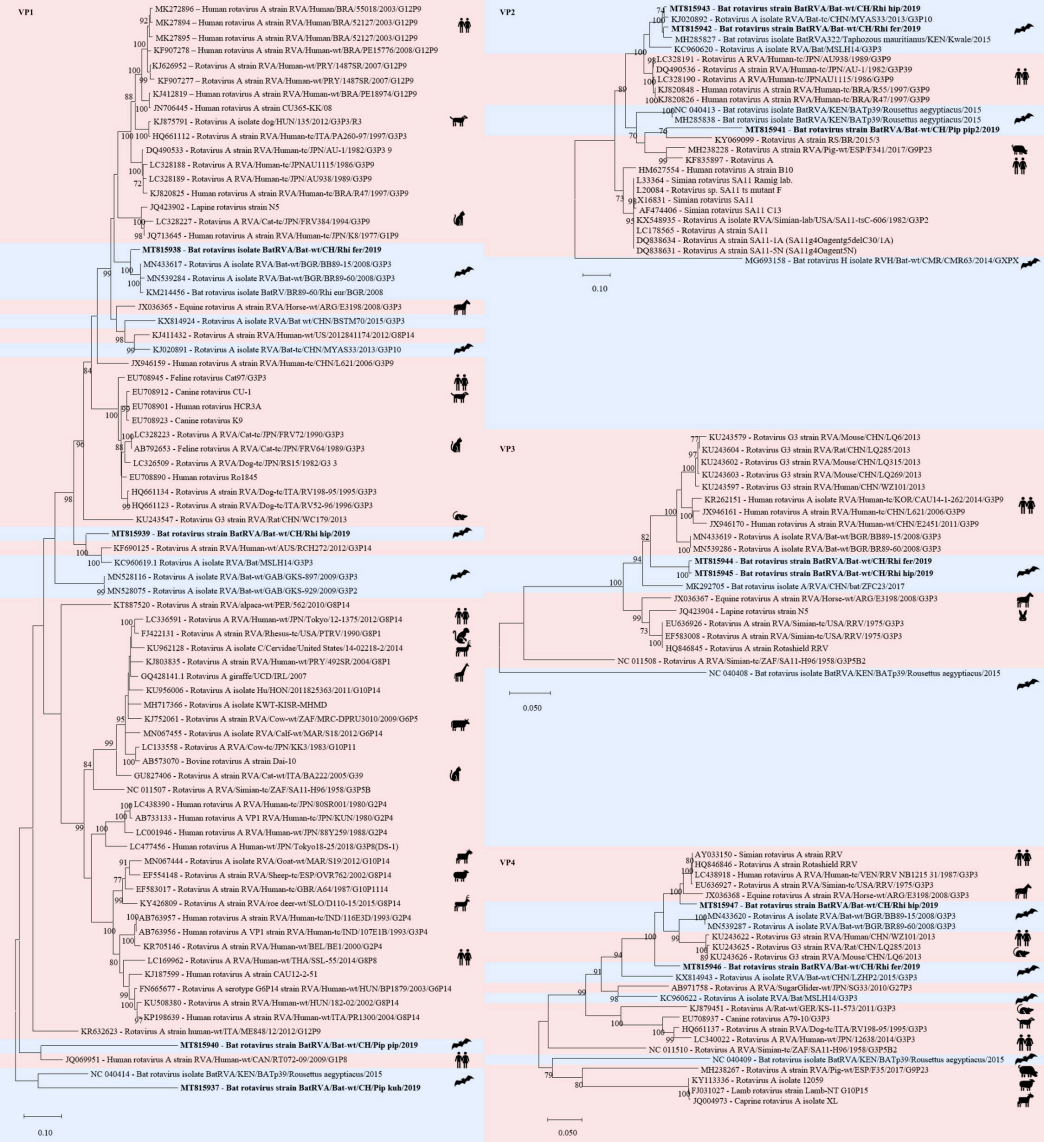
Phylogenetic analysis of specific regions of the rotavirus A genome. The sequences obtained in this study (GenBank acc. numbers MT815937-47) are shown in bold. Sequences of non-bat associated viruses are marked with a red background and those of bat associated viruses with a blue background. The pictograms on the right side represent the species in which the virus was detected. Sequences were aligned using Muscle. For phylogenetic analysis, the Maximum likelihood tree with 1’000 bootstraps was used. Only values ≥ 70% are displayed.

Furthermore, in five samples 11 contigs were assemble to RVA, each covering one to two segments of VP1 to VP4 (S4 Table). The samples included a ground stool sample of a *Rhinolophus hipposiderus* colony and a *Rhinolophus ferrumequinum* colony, two intestine pools of *Pipistrellus pipistrellus* and one combined liver/spleen pool of *Pipistrellus kuhlii*. Three contigs (2’8328 bp, 1’617bp, 2’687 bp) were 96% or 73% similar to a bat RVA VP2 segment (GenBank acc. number KJ020892) or a human RVA VP2 segment (GenBank acc. number KF835897), respectively. Four contigs had 74 to 95% nt similarity to different RVA VP1 segments from bats, humans, and pigs (GenBank acc. number MN433617; MH238214; KF690125; EF583033) (Fig 8).

#### Parvoviridae

**Fig 9:**
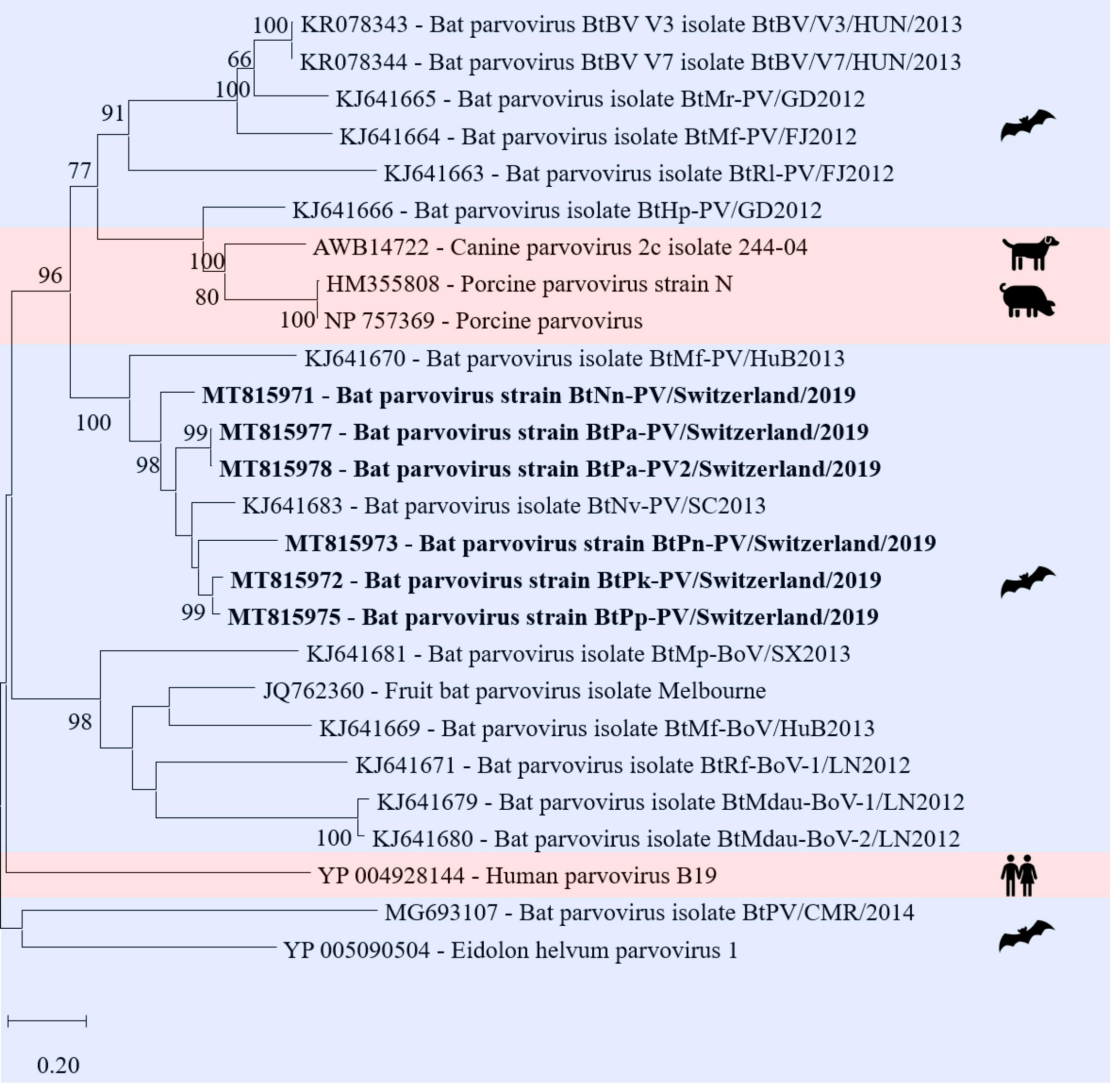
Phylogenetic analysis of the NS1 region of parvoviruses. The sequences obtained in this study (GenBank acc. numbers MT815971-73, MT815975, 77, 78) are shown in bold. Sequences of non-bat associated viruses are marked with a red background and those of bat associated viruses with a blue background. The pictograms on the right side represent the species in which the virus was detected. Sequences were aligned using Muscle. For phylogenetic analysis, the Maximum likelihood tree with 1’000 bootstraps was used. Only values ≥ 70% are displayed.

*Parvoviridae* are non-enveloped viruses with a ssDNA genome of either positive or negative polarity. The virion has an icosahedral morphology and a size of 21–26 nm. The genome consists of one linear DNA segment of 4 to 6.3 kb [74]. In total, nine contigs with lengths between 746 and 2’908 nt from different samples and bat species were assembled to one bat PV genome (GenBank acc. number KJ641683) with nt similarity between 74 and 85% (Fig 9) belonging to the subfamily *Parvovirinae* and are unclassified *Parvovirinae*. The contigs covered the ORFs coding for the nonstructural protein 1 (NS1) and viral protein 1 (VP1) (S4 Table). The nine contigs originated from an intestine pool of *Pipistrellus pipistrellus*, an intestine pool of *Plecotus auritus*, a fecal pool of *Nyctalus noctula*, two fecal pools of *Pipistrellus nathusii*, a combined liver/spleen pool of *Pipistrellus kuhlii*, and a combined liver/spleen pool of *Plecotus auritus*.

#### Circoviridae

**Fig 10:**
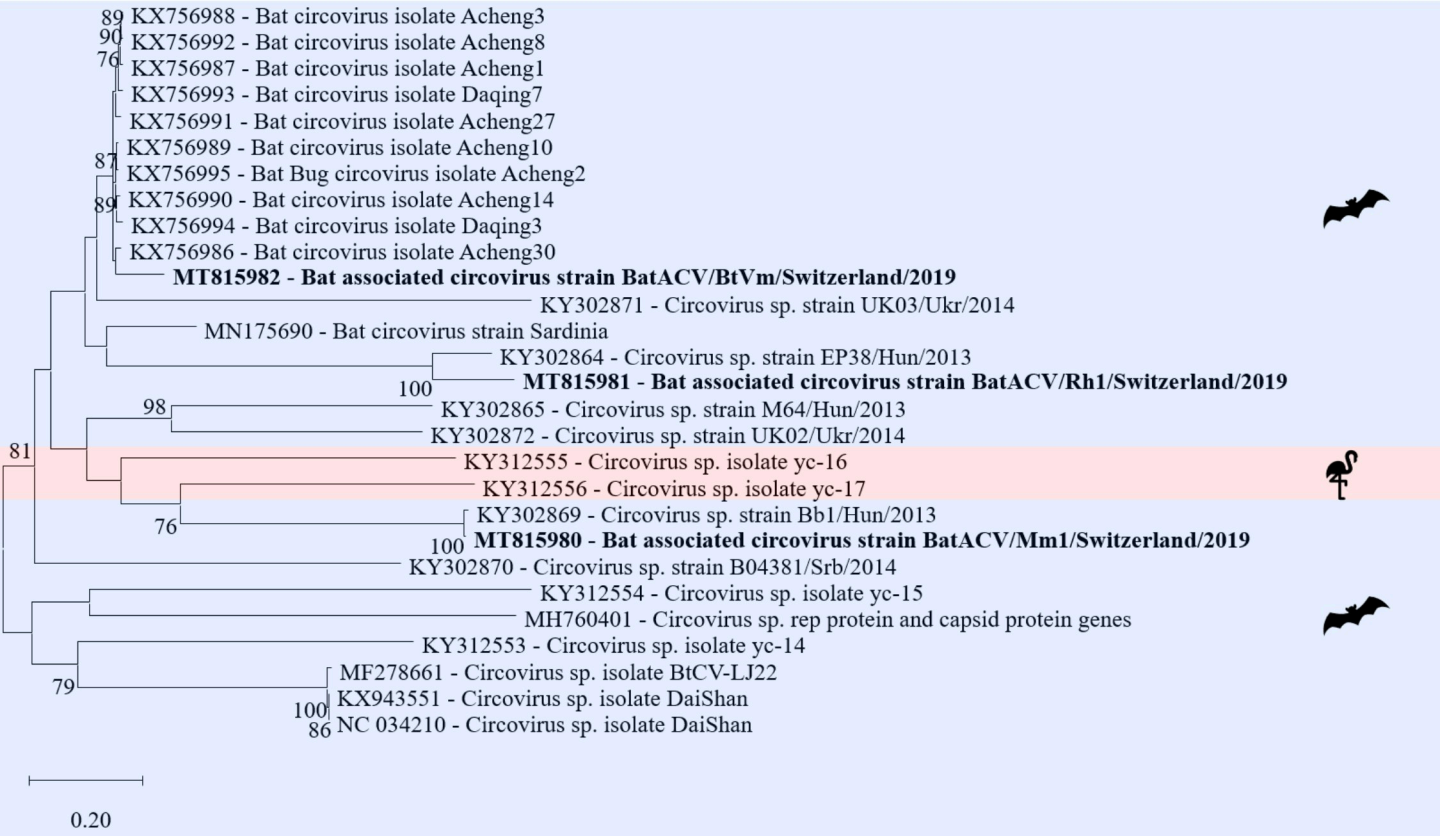
Phylogenetic analysis of the full genome of circoviruses. The sequences obtained in this study (GenBank acc. numbers MT815980-82) are shown in bold. Sequences of non-bat associated viruses are marked with a red background and those of bat associated viruses with a blue background. The pictograms on the right side represent the species in which the virus was detected. Sequences were aligned using Muscle. For phylogenetic analysis, the Maximum likelihood tree with 1’000 bootstraps was used. Only values ≥ 70% are displayed.

*Circoviridae* was the most abundant vertebrate virus family in this study (84% of all reads assembled to vertebrate viruses). *Circoviridae* are small non-enveloped viruses with a ssDNA genome of either positive or negative polarity. The virion has an icosahedral morphology and a size of 12–27 nm. The genome is a circular DNA with a length of 1.7–2.3 kb. These viruses infect mainly vertebrate hosts [74]. Three samples revealed full genomes, including the two ORFs coding for the replicase and the capsid protein (S4 Table) of bat-associated circoviruses belonging to the family *Circoviridae* and unclassified Circoviruses. In one fecal sample of *Vespertilio murinus* a contig with a length of 2’134 nt and 90% nt similarity to a bat circovirus (GenBank acc. number KX756991) from China was assembled (Fig 10). Two contigs with lengths of 1’641 bp and 1’668 nt, originating from a brain sample of a *Myotis myotis* bat, a ground stool sample of a *Myotis myotis* colony and a ground stool sample of a *Rhinolophus hipposideros* colony, with 99% nt similarity to two different circovirus sp. (GenBank acc. number KY302869; KY302864) from Hungary were assembled (Fig 10).

#### Hepeviridae

*Hepeviridae* are small non-enveloped viruses with an icosahedral capsid of 27–34 nm. The genome consists of a linear, positive sensed ssRNA of 6.6 to 7.2 kb. *Hepeviridae* have so far been detected only in vertebrate hosts [74].

**Fig 11:**
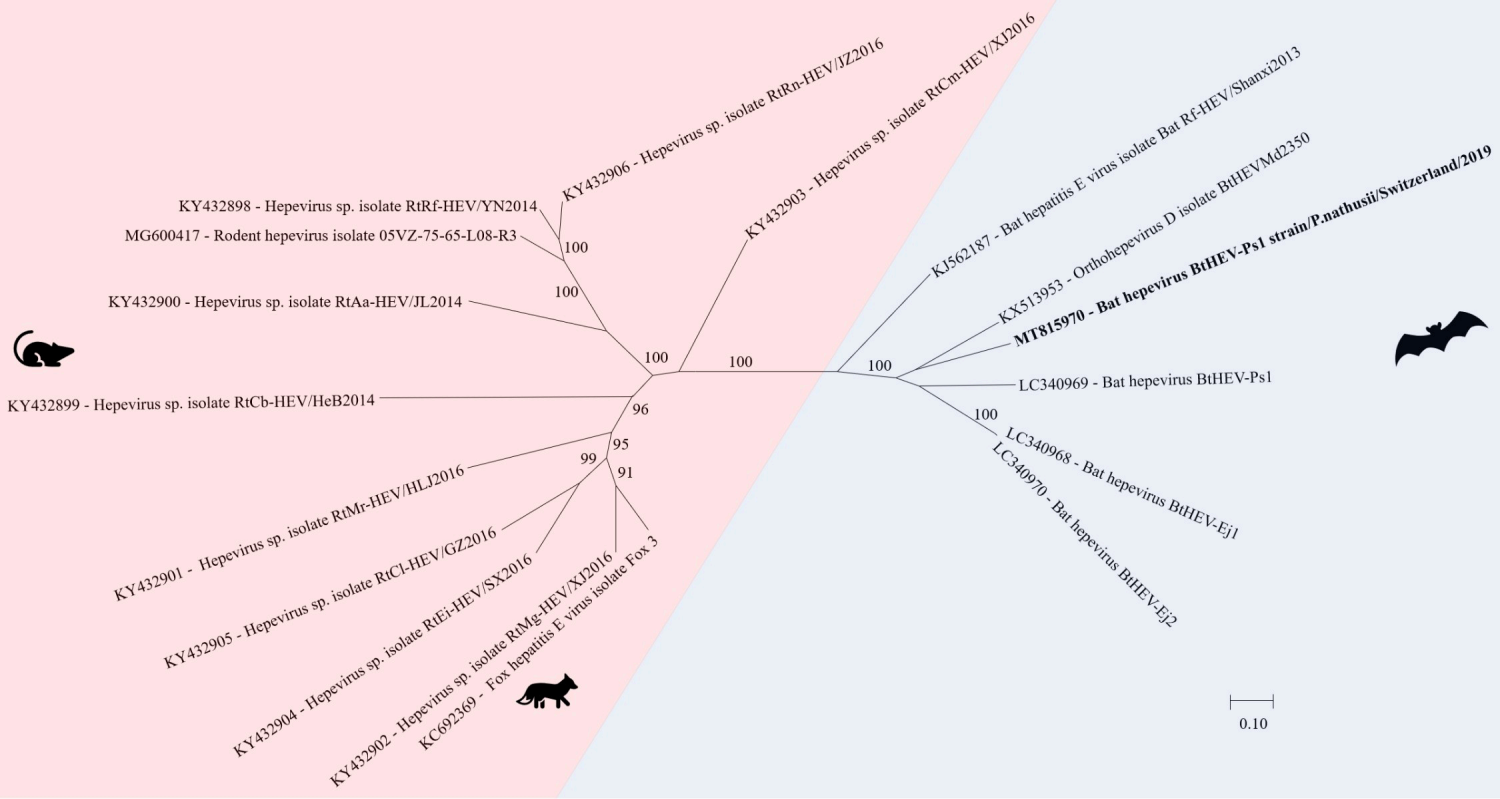
Phylogenetic analysis of full and partial genome of hepeviruses. The sequence obtained in this study (GenBank acc. numbers MT815970) is shown in bold. Sequences of non-bat associated viruses are marked with a red background and those of bat associated viruses with a blue background. The pictograms on the right side represent the species in which the virus was detected. Sequences were aligned using Muscle. For phylogenetic analysis, the Maximum likelihood tree with 1’000 bootstraps was used. Only values ≥ 70% are displayed.

In a fecal pool of *Pipistrellus nathusii*, an orthohepevirus D contig with a length of 6’647 nt was assembled and showed 75% nt similarity to a bat hepevirus genome from China (GenBank acc. number KX513953) belonging to the genus orthohepevirus. The contig covered two ORF’s coding for the nonstructural polyprotein and the capsid protein (S4 Table). In the phylogenetic analysis of Fig 11, the separate clade of bat hepevirus is shown.

## Discussion

This study included samples from 7’291 bats of 18 different species. Specifically, organ or fecal samples from 245 individual animals and ground stool samples from 36 different bat colonies (8 different species) with approximately 7’046 individual animals were analyzed by NGS. This metagenomic analysis revealed sequences of a total of 39 different virus families including 16 virus families of vertebrates (Fig 1A and 1B). Similar viral metagenomic studies of bats from Singapore, China, and Myanmar have yielded comparable virus diversities [6, 60, 78, 79]. However, direct comparison of the virus genome diversity of bats in Switzerland with those from other countries is difficult because of different sample types, sample sizes, species, pooling strategies, and health conditions of the animals. Furthermore, due to different sample preparation methods as well as pipelines used for analysis the output may introduce bias to the final results. Since this is the first pilot study on the virome of bats from Switzerland, a large number of different bat species and samples were included in order to obtain a first overview. In Croatia, the virome of 455 bats from seven bat species (*Myotis myotis*, *Miniopterus schreibersii*, *Rhinolophus ferrumequinum*, *Eptesicus serotinus*, *Myotis blythii*, *Myotis nattereri* and *Myotis emarginatus*) was determined from saliva, fecal and guano samples, and showed a dominance of viruses from invertebrates [80], similar to our study. Moreover, as reported previously the potential zoonotic viruses in European bats include viruses from the families *Adenoviridae*, *Astroviridae*, *Coronaviridae*, *Hepeviridae*, *Reoviridae* and *Retroviridae* [5, 32, 81–97], which have all been detected in our study as well. Interestingly, the data obtained in a study conducted in France is strikingly different. While the number of different virus families of vertebrates detected was much smaller (8), it included many virus families that were not detected in our study, such as *Bunyaviridae*, *Flaviviridae*, *Herpesviridae*, and *Orthomyxoviridae* [5]. These differences may be due to the different populations sampled in that study (bats with behavioral changes and close human contact) compared to our study (mainly healthy bats) as well as the larger numbers of different bat species (18) and sample types (fecal samples, liver, spleen, lung, brain and intestine) investigated in our study.

The question whether native bat species harbor CoVs was of special interest in the present investigation. Such virus sequences were indeed detected and belonged to either the alpha- or betacoronaviruses while no gamma- or deltacoronaviruses were detected in the samples. This finding was consistent with the results of previous studies in other geographical locations [32, 40, 48, 54, 89, 98, 99] and the common knowledge about host preferences of the different coronaviruses; alpha- and betacoronaviruses mainly infect mammals including humans while the gamma- and deltacoronaviruses infect birds [6, 100]. However, so far none of the coronaviruses detected in European bats were 100% identical to human pathogenic viruses such as SARS-CoV-1, MERS-CoV and SARS-CoV-2 [32]. Nevertheless, closely related CoVs have been detected i.e., in Italy, where the genomes of two viruses closely related to MERS-CoV (80% nt similarity [89]) have been detected in *Pipistrellus pipistrellus* and *Hypsugo savii*. Similarly, in a ground stool sample of a *Vespertilio murinus* colony sampled in our study, a contig of 20 kb with a nt similarity of 86% to a MERS-like CoV genome from China was assembled [101].

Rhabdovirus sequences were not identified in our study, although rabies lyssaviruses have previously been detected in *Myotis daubentonii* bats in Switzerland, albeit at low prevalence (0.36%) [38]. Rhabdoviruses, especially viruses from the genus Lyssavirus, are zoonoses. In Europe, several lyssaviruses were detected in bats including the European bat lyssavirus 1 and 2 (EBLV-1/2). It is known that bats can transmit rabies/lyssaviruses to humans by biting and scratching [32]. In the present study neither EBLV-1 nor EBLV-2 genomes were detected. *Eptesicus serotinus* and *Myotis daubentonii*, both endemic in Switzerland, are known to be the main hosts of EBLV-1 or 2, respectively [7, 32]. However, in our study only samples of *Myotis daubentonii* were collected.

In Switzerland, five bat species, *Nyctalus leisleri*, *N*. *noctula*, *N*. *lasiopterus*, *Pipistrellus nathusii and Vespertilio murinu*s, migrate several hundred kilometers between summer and winter quarter [7]. Migration habits may lead to harboring more viruses due to broader contact with various surroundings, e.g. other bat populations, other living areas, and insects. However, our data reveal that only in two migrating species i.e., *Pipistrellus nathusii* and *Vespertilio murinus*, the number of different virus families detected, 16 and 10, respectively, was higher than the average 9.6 virus families per bat species. The observation that the virome of migrating bats is not more diverse than that of non-migrating bats has been made in previous studies as well [16, 17, 102, 103].

In the present study, 90% of all assembled reads belonged to virus families of non-vertebrates. This observation is not consistent with other reports where vertebrate viruses clearly dominated the virome [5, 6, 42, 43, 60, 61, 104]. This difference may be explained by the different sample material used, only feces or only tissue samples or both as in our study [5, 6, 42, 43, 60, 61, 104]. The viromes of bat guano from Texas, California, and Singapore consisted of mainly non-vertebrate viruses, particularly insect viruses, thereby supporting the hypothesis that dietary habits are a likely explanation for the large numbers of insect viruses detected in fecal samples [42–44, 60, 104]. Although most of our samples were pooled organs, more reads were assembled to non-vertebrate than vertebrate viruses, since all sampled bat species were insectivorous, and because a higher number of reads was obtained from fecal pools. Another interesting observation was the high number of reads assembled to honeybee viruses in fecal pools of *Myotis myotis* colonies. Bee-associated viruses were found also in different bat species from other countries [42–44, 104]. The detected sequences were mostly related to the deformed wing virus (*Varrora destructor* virus) which causes either asymptomatic infection in western honeybees (*Apis mellifera*) or can lead to wing deformity, behavioral changes, and early mortality in other bee species [42, 105].

Adenoviruses have been detected in bats from several countries including Italy, Germany, China, and Hungary, however, the number of different adenoviruses detected in bats is small [36, 40, 81, 82, 106, 107]. The genus *Mastadenovirus* has been thought to infect only mammals, including humans [36, 108, 109]. However, cross-species transmission has nevertheless been observed with adenoviruses, and circulation between different animal species and transmission from animals to humans is possible [36, 110, 111].

Most metagenomic studies of bats use feces as sample material. As *Parvoviridae* can be detected in high concentrations in human blood samples [112], Canuti et al. sampled EDTA-blood of two bat species and showed a relatively low prevalence of PVs in bats from West Africa and Central America [39]. In our study nearly a quarter of the samples had reads assembled to *Parvovirinae*, mainly bat PVs. For the emergence of new viruses, their evolutionary potential and mutation rate is of particular importance. RNA viruses are known to mutate much more frequently than DNA viruses, however, among the DNA viruses the PVs have a relatively high mutation rate, and host switching has been observed [113, 114]. On the other hand, PVs are known to be coevolving with their host [115].

*Circoviridae* was the most abundant vertebrate virus family in this study (84%) while in other studies it was the *Parvoviridae* [6]. In addition, the prevalence of circovirus genomes in this study was higher than that revealed in previous reports [40, 44, 116, 117].

RVA and RVH have been detected in metagenomic analyses of bat samples from different geographical locations [75–77]. However, it is not known whether RV infections in bats lead to disease [76, 118]. RVs have been detected also in several other wild animals [119].

Bats are known hosts for different hepeviruses of the genus *Orthohepevirus* [96, 120, 121]. Bat hepeviruses, including the contig revealed in this study, seem to cluster with one another and generate a separate clade withing the *Orthohepeviruses*, the Orthohepevirus D [96, 120]. This indicates that bats may not serve as reservoirs for hepeviruses infecting other mammals including humans [96].

The most interesting finding in this study was an almost complete genome of a MERS-like CoV detected in a ground stool sample of a *Vespertilio murinus* colony. It will be interesting to study the zoonotic potential of this bat betacoronavirus and to monitor the colony in which it was detected over time as this would allow to assess the accumulation of mutations in the CoV genome in a natural reservoir host.

Human interaction with wildlife is one of the major contributors to zoonotic spillover and this impact should be reassessed [57]. Metagenomic analysis of ground stool samples of bat colonies represents an ideal non-invasive high throughput method to monitor the complexity of the viral genome diversity. It allows also to detect viruses with zoonotic potential and to assess the potential risk for their transmission to other species including humans. This first assessment of the virome of Swiss bats forms a platform for future in-depth studies to investigate changes in virus prevalence, virus biology, virus-host interaction, and virus emergence.

## Supporting information

S1 Fig Locations in Switzerland where samples were collected, and number of animals sampled. (TIF)

S2 Fig Viral reads detected of viruses from vertebrates and non-vertebrates shown as percentage of the total number of reads generated from pools of each bat species. Numbers in parentheses represent the numbers of virus families from vertebrates/non-vertebrates; *migrating bat species.
(TIF)

S1 Table Ground stool samples of colonies. 7’046 animals of 8 different bat species and from 36 different bat colonies were sampled.
(DOCX)

S2 Table Fecal samples of individual animals. 137 individual animals from 16 bat species were sampled.
(DOCX)

S3 Table Tissue samples of individual animals. 108 individual animals were dissected and the lung, liver combined with spleen, intestine and brain collected.
(DOCX)

S4 Table Overview of the genome organization of the contigs from de novo analysis. (DOCX)
