# Supplementary figures and images for: Metagenomic analysis of fecal and tissue samples from 18 endemic bat species in Switzerland revealed a diverse virus composition including potentially zoonotic viruses
Source: PLoS One. 2021 Jun 16;16(6):e0252534. doi: 10.1371/journal.pone.0252534 (PMC8208571; doi:10.1371/journal.pone.0252534)

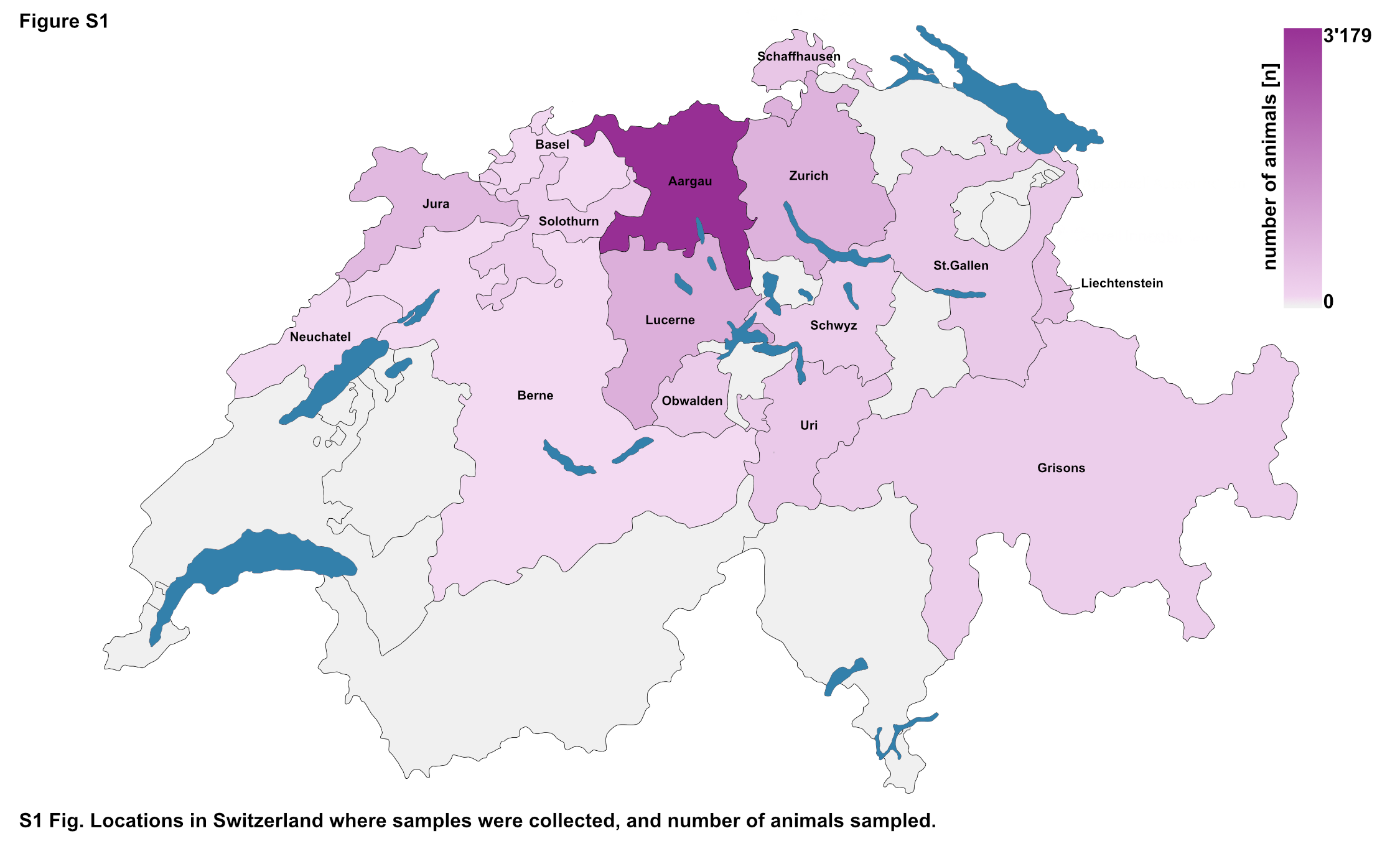

Supplement: S1 Fig — (TIF) [file pone.0252534.s001.tif]

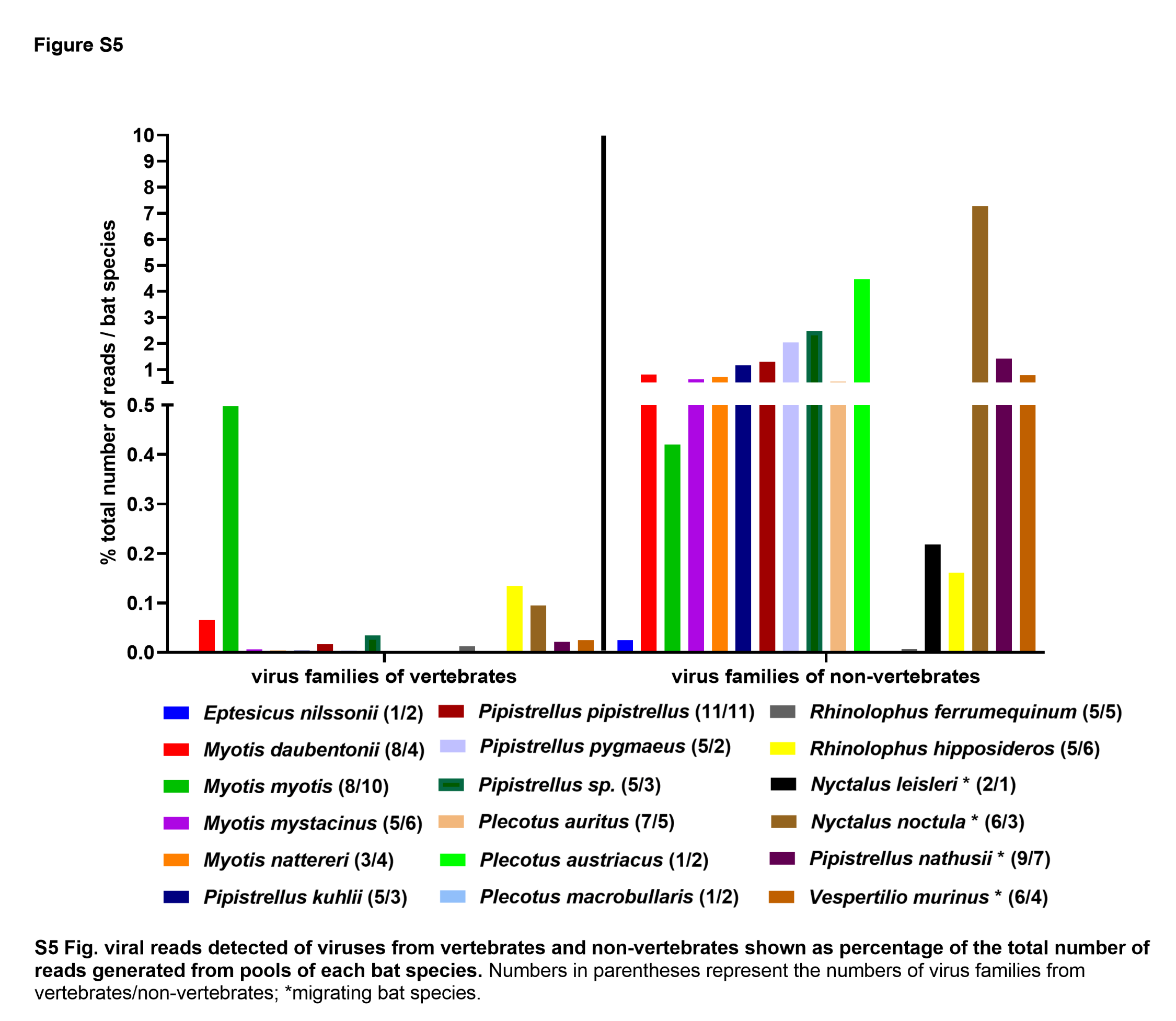

Supplement: S2 Fig — Numbers in parentheses represent the numbers of virus families from vertebrates/non-vertebrates; *migrating bat species. (TIF) [file pone.0252534.s002.tif]
