# Supplementary material for: Metagenomic analysis of fecal and tissue samples from 18 endemic bat species in Switzerland revealed a diverse virus composition including potentially zoonotic viruses
Source: PLoS One. 2021 Jun 16;16(6):e0252534. doi: 10.1371/journal.pone.0252534 (PMC8208571; doi:10.1371/journal.pone.0252534)
Supplement: S1 Table — 7’046 animals of 8 different bat species and from 36 different bat colonies were sampled. (DOCX) [file pone.0252534.s003.docx]

**S1 Table. Ground stool samples of colonies.** 7’046 animals of 8 different bat species and from 36 different bat colonies were sampled.

|  | **Bat species** | ***Myotis myotis*** | ***Vespertilio murinus*** | ***Myotis daubentonii*** | ***Myotis mystacinus*** | ***Myotis nattereri*** | ***Rhinolophus ferrumequinum*** | ***Rhinolophus hipposideros*** | ***Nyctalus noctula*** | **Total** |
| --- | --- | --- | --- | --- | --- | --- | --- | --- | --- | --- |
| **Canton** |  |  |  |  |  |  |  |  |  |  |
| **Aargau** | number of animals | 2’454 | 200 | 200 | 50 | 50 | 5 |  | 200 | 3’159 |
|  | number of colonies | 7 | 1 | 1 | 1 | 1 | 1 |  | 1 | 13 |
| **Grisons** | number of animals |  |  |  |  |  | 186 | 12 |  | 198 |
|  | number of colonies |  |  |  |  |  | 1 | 1 |  | 2 |
| **Jura** | number of animals | 600 |  |  |  |  |  |  |  | 600 |
|  | number of colonies | 2 |  |  |  |  |  |  |  | 2 |
| **Lucerne** | number of animals | 778 |  |  |  |  |  |  |  | 778 |
|  | number of colonies | 4 |  |  |  |  |  |  |  | 4 |
| **Obwalden** | number of animals | 250 |  |  |  |  |  |  |  | 250 |
|  | number of colonies | 1 |  |  |  |  |  |  |  | 1 |
| **Schaffhausen** | number of animals | 370 |  |  |  |  |  |  |  | 370 |
|  | number of colonies | 3 |  |  |  |  |  |  |  | 3 |
| **Schwyz** | number of animals | 212 |  |  |  |  |  |  |  | 212 |
|  | number of colonies | 2 |  |  |  |  |  |  |  | 2 |
| **Solothurn** | number of animals | 208 |  |  |  |  |  |  |  | 208 |
|  | number of colonies | 2 |  |  |  |  |  |  |  | 2 |
| **St. Gallen** | number of animals | 310 |  |  |  |  |  |  |  | 310 |
|  | number of colonies | 1 |  |  |  |  |  |  |  | 1 |
| **Uri** | number of animals | 300 |  |  |  |  |  |  |  | 300 |
|  | number of colonies | 1 |  |  |  |  |  |  |  | 1 |
| **Zurich** | number of animals | 568 |  |  |  |  |  |  |  | 568 |
|  | number of colonies | 4 |  |  |  |  |  |  |  | 4 |
| **Lichtenstein** | number of animals | 93 |  |  |  |  |  |  |  | 93 |
|  | number of colonies | 1 |  |  |  |  |  |  |  | 1 |
| **Total number of animals** |  |  |  |  |  |  |  |  |  | **7’046** |
| **Total number of colonies** |  |  |  |  |  |  |  |  |  | **36** |
