# Supplementary material for: Metagenomic analysis of fecal and tissue samples from 18 endemic bat species in Switzerland revealed a diverse virus composition including potentially zoonotic viruses
Source: PLoS One. 2021 Jun 16;16(6):e0252534. doi: 10.1371/journal.pone.0252534 (PMC8208571; doi:10.1371/journal.pone.0252534)
Supplement: S2 Table — 137 individual animals from 16 bat species were sampled. (DOCX) [file pone.0252534.s004.docx]

**S2 Table. Fecal samples of individual animals.** 137 individual animals from 16 bat species were sampled.

| **Canton** | **Bat species** | **Number of animals** | **Number of samples** |
| --- | --- | --- | --- |
| **Aargau** | *Pipistrellus nathusii* | 4 | 1 |
|  | *Pipistrellus kuhlii* | 1 | 1 |
|  | *Pipistrellus sp.* | 1 | 1 |
| **Basel** | *Pipistrellus pipistrellus* | 1 | 1 |
| **Lucerne** | *Myotis daubentonii* | 1 | 1 |
|  | *Myotis myotis* | 2 | 1 |
|  | *Pipistrellus nathusii* | 1 | 1 |
|  | *Pipistrellus pygmaeus* | 1 | 1 |
| **Neuchatel** | *Myotis myotis* | 2 | 1 |
|  | *Pipistrellus nathusii* | 2 | 1 |
|  | *Pipistrellus pipistrellus* | 2 | 1 |
| **Schaffhausen** | *Plecotus austriacus* | 1 | 1 |
| **Zurich** | *Myotis daubentonii* | 4 | 1 |
|  | *Myotis myotis* | 1 | 1 |
|  | *Myotis mystacinus* | 1 |  |
|  | *Nyctalus noctula* | 72 | 11 |
|  | *Pipistrellus kuhlii* | 8 | 1 |
|  | *Pipistrellus nathusii* | 7 | 1 |
|  | *Pipistrellus pipistrellus* | 13 | 2 |
|  | *Pipistrellus sp* | 7 | 1 |
|  | *Plecotus aurtius* | 4 | 2 |
|  | *Vespertilio murinus* | 1 | 1 |
|  | **Total number of animals/ pools** | **137** | **33** |
