# Supplementary material for: Metagenomic analysis of fecal and tissue samples from 18 endemic bat species in Switzerland revealed a diverse virus composition including potentially zoonotic viruses
Source: PLoS One. 2021 Jun 16;16(6):e0252534. doi: 10.1371/journal.pone.0252534 (PMC8208571; doi:10.1371/journal.pone.0252534)
Supplement: S3 Table — 108 individual animals were dissected and the lung, liver combined with spleen, intestine and brain collected. (DOCX) [file pone.0252534.s005.docx]

**S3 Table. Tissue samples of individual animals.** 108 individual animals were dissected and the lung, liver combined with spleen, intestine and brain collected.

| **Canton** | **Bat species** | **Number of animals** | **Number of lung; liver/spleen; intestine; brain samples** | **Number of pools** |
| --- | --- | --- | --- | --- |
| **Aargau** | *Myotis myotis* | 11 | 11; 11; 11; 11 | 14 |
|  | *Myotis mystacinus* | 2 | 2; 2;2;0 | 3 |
|  | *Pipistrellus pipistrellus* | 1 | 1; 1; 1;0 | 3 |
| **Basel** | *Pipistrellus kuhlii* | 7 | 7; 7; 7; 0 | 3 |
|  | *Pipistrellus nathusii* | 10 | 10; 10; 10; 0 | 3 |
|  | *Pipistrellus pipistrellus* | 16 | 16; 16; 16; 0 | 3 |
| **Berne** | *Plecotus auritus* | 1 | 1; 1; 1;0 | 3 |
| **Grisons** | *Eptesicus nilssonii* | 3 | 3; 3; 3; 0 | 3 |
|  | *Myotis nattereri* | 1 | 3; 3; 3; 0 | 3 |
|  | *Nyctalus leisleri* | 2 | 2; 2;2;0 | 3 |
|  | *Pipistrellus nathusii* | 1 | 1; 1; 1;0 | 3 |
|  | *Pipistrellus pipistrellus* | 2 | 2; 2;2;0 | 3 |
|  | *Plecotus auritus* | 2 | 2; 2;2;0 | 3 |
|  | *Plectous macrobullaris* | 1 | 1; 1; 1;0 | 3 |
| **Jura** | *Pipistrellus pipistrellus* | 1 | 1; 1; 1;0 | 3 |
| **Lucerne** | *Myotis myotis* | 1 | 1; 1; 1;0 | 3 |
|  | *Pipistrellus pygmaeus* | 1 | 1; 1; 1;0 | 3 |
| **Neuchatel** | *Pipistrellus pipistrellus* | 1 | 1; 1; 1;0 | 3 |
| **St. Gallen** | *Plecotus auritus* | 1 | 1; 1; 1;0 | 3 |
|  | *Pipistrellus kuhlii* | 1 | 1; 1; 1;0 | 3 |
| **Zurich** | *Myotis daubentonii* | 3 | 3; 3; 3; 0 | 3 |
|  | *Myotis mystacinus* | 1 | 1; 1; 1;0 | 3 |
|  | *Pipistrellus kuhlii* | 11 | 11; 11; 11;0 | 6 |
|  | *Pipistrellus nathusii* | 5 | 5; 5; 5; 0 | 6 |
|  | *Pipistrellus pipistrellus* | 14 | 14; 14; 14; 1 | 7 |
|  | *Pipistrellus sp* | 4 | 4; 4; 4; 0 | 3 |
|  | *Plecotus auritus* | 2 | 2; 2;2;0 | 3 |
|  | *Vespertilio murinus* | 2 | 2; 2;2;0 | 3 |
|  | **Total** | **108** | **342** | **105** |
