# Supplementary material for: Metagenomic analysis of fecal and tissue samples from 18 endemic bat species in Switzerland revealed a diverse virus composition including potentially zoonotic viruses
Source: PLoS One. 2021 Jun 16;16(6):e0252534. doi: 10.1371/journal.pone.0252534 (PMC8208571; doi:10.1371/journal.pone.0252534)
Supplement: S4 Table — (DOCX) [file pone.0252534.s006.docx]

**S4 Table. Overview of the genome organization of the contigs from de novo analysis.**

| Virus | GenBank acc. number | Strain name | Protein/ ORF | Position (nt) | Number of amino acids (nt) | Reference sequence |
| --- | --- | --- | --- | --- | --- | --- |
| Adenovirus |  |  |  |  |  |  |
|  | MT815927 | Bat adenovirus 2 strain PNV1/Switzerland/2019 hexon, protease, DNA-binding protein, 100K, 22k, pVIII, 12.5K, E3 region, fiber, U exon protein, hypothetical protein, 34K, ORFD, C, B, A genes, complete cds | Hexon  Protease  DNA-binding protein  100K  22K  pVIII  12.5K  E3 region/ ORF1  Fiber  U exon protein  Hypothetikal protein/ ORF6/7  34K  Hypothetical protein/ORF D  Hypothetical protein/ORF C  Hypothetical protein/ORF B  Hypothetical protein/ORF A | 155 – 2’878  2’890 – 3’510  4’939 – 3’554  4’951 – 7’038  6’875 – 7’405  7’548 – 8’237  8’224 – 8’574  8’597 – 9’769  9’963 – 11’636  9’964 – 9’797  11’890 – 11’651  12’676 – 11’891  13’129 – 12’677  13’418 – 13’005  13’804 – 13’445  14’287 – 13’892 | 2’723  620  1’385  2’087  530  689  350  1’172  1’673  167  239  785  452  413  359  395 | [1-3], NC_015932, NC_016895, NC_031948 |
|  | MT815928 | Bat adenovirus 2 strain PNV1/Switzerland/2019 E1A, E1B 19K, E1B 55K, IX, IVa2, DNA polymerase, pTP, 52K, pIIIa and III genes, complete cds; pVII gene, partial cds | E1A  E1B 19K  E1B 55K  IX  IVa2  DNA polymerase  pTP  52K  pIIIa  III  pVII | 999 – 1’208  1’377 – 1’916  1’739 – 3’073  3’140 – 3’451  4’665 – 3’448  7’754 – 4’557  9’685 – 7’820  9’680 – 10’873  10’746 – 12’491  12’560 – 13’993  14’025 - >14’108 | 209  539  1’334  311  1’217  3’197  1’865  1’193  1’745  1’433  > 83 | [1-3]  NC_015932, NC_016895, NC_031948 |
|  | MT815929 | Bat adenovirus 2 strain PPV2/Switzerland/2019 E1A, E1B 19K, E1B 55K, IX, IVa2, DNA polymerase, pTP, 52K and pIIIa genes, complete cds; III gene, partial cds | E1A  E1B 19K  E1B 55k  IX  IVa2  DNA polymerase  pTP  52K  pIIIa  III | 826 – 1’035  1’204 – 1’749  1’566 – 2’906  2’973 – 3’284  4’498 – 3’281  7’587 – 4’390  9’518 – 7’653  9’513 – 10’706  10’579 – 12’327  12’396 - >13’696 | 209  545  1’340  311  1’217  3’197  1’188  1’193  1’748  > 1’300 | [1-3]  NC_015932, NC_016895, NC_031948 |
|  | MT815930 | Bat adenovirus 2 strain PPV2/Switzerland/2019 hexon, protease and DNA-binding portein genes, complete cds; 100K gene partial cds | Hexon  Protease  DNA-binding protein  100K | 7 – 699  711- 1’331  2’760 – 1’375  2’772 - >3’125 | 692  620  1’385  > 353 | [1-3]  NC_015932, NC_016895, NC_031948 |
|  | MT815931 | Bat adenovirus 2 strain PPV2/Switzerland/2019, III and pVII genes, complete cds; V gene, partial cds | III  pVII  V | 103 – 1’056  1’088 – 1’465  1’533 – > 2’184 | 953  377  > 651 | [1-3]  NC_015932, NC_016895, NC_031948 |
|  | MT815932 | Bat adenovirus 2 strain PPV2/Switzerland/2019, 100K, 22K, pVIII, 12.5K, E3 region and U hexon genes, complete cds; fiber gene, partial cds | 100K  22K  pVIII  12.5K  E3 region/ORF1  Fiber  U exon protein | 25 – 1’725  1’562 – 2’092  2’235 – 2’924  2’911 – 3’261  3’284 – 4’456  4’650 - > 4’953  4’651 – 4’484 | 1’700  530  689  350  1’172  > 303  167 | [1-3]  NC_015932, NC_016895, NC_031948 |
|  | MT815933 | Bat adenovirus isolate Nynoc/Switzerland/2019 DNA polymerase (pol) gene, partial cds | DNA polymerase | 31- > 1’673 | > 1’642 | [1-3]  NC_015932, NC_016895, NC_031948 |
|  | MT815934 | Bat mastadenovirus Pnathusii/Switzerland/2019 E1B, IVa2, DNA polymerase and DNA terminal protein genes, complete cds; 52K gene, partial cds | Small T-antigen/ E1B protein  Large T-antigen/ E1B protein  IVa2  IVa2  DNA polymerase  DNA terminal protein  52K | 643 – 1‘095  996 – 2‘225  3‘311 – 2‘577  3‘596 – 3‘366  6‘775 – 3‘650  8‘607 – 6‘937  8‘772- > 9‘191 | 452  771  734  230  3‘125  1‘670  419 | [1-3]  NC_015932, NC_016895, NC_031948 |
|  | MT815935 | Bat mastadenovirus B strain Ppipistrellus/Switzerland/2019 hypothetical protein CaV1gp02, E1B 19K, E1B 55K, IX, IV2a, DNA polymerase and pTP genes, complete cds | Hypothetical protein CaV1gp02  E1B 19K  E1B 55k  IX  IV2a  DNA polymerase  pTP | 195 – 398  557 – 1’084  919 – 2’256  2’327 – 2’653  3’867 – 2’650  6’968 – 3’759  8’863 – 7’034 | 203  527  1’337  326  1’217  3’209  1’829 | [1-3]  NC_015932, NC_016895, NC_031948 |
|  | MT815936 | Bat mastadenovirus B strain Ppipistrellus/Switzerland/2019, pX and pVI genes, complete cds; hexon gene, partial cds | pX  pVI  hexon | 350 – 559  613 – 1’320  1’388 - > 3’570 | 209  707  > 2’182 | [1-3]  NC_015932, NC_016895, NC_031948 |
|  |  |  |  |  |  |  |
| Circovirus |  |  |  |  |  |  |
|  | MT815980 | Bat associated circovirus strain BatACV/Mm1/Switzerland/2019, complete genome | Replicase  Capsid | <1 – 885  1’523 - 900 | > 885  623 | [4, 5] NC_034210 |
|  | MT815981 | Bat associated circovirus strain BatACV/Rh1/Switzerland/2019, complete genome | Capsid  Replicase | 4 – 633  1’664 - 789 | 629  875 | [4, 5] NC_034210 |
|  | MT815982 | Bat associated circovirus strain BatACV/BtVm/Switzerland/2019, complete genome | Relicase  Capsid | 54 – 974  2’044 – 1’265 | 920  779 | [4, 5] NC_034210 |
|  |  |  |  |  |  |  |
| Coronavirus |  |  |  |  |  |  |
|  | MT818221 | Middle East respiratory syndrome-related coronavirus strain Bat CoV/V.murinus/Switzerland/2019, ORF1ab (orf1ab), ORF1a (orf1ab) and S protein genes, partial cds | ORF1a  ORF1ab  S protein | 43 – 10’482  10’671 – 18’545  18’487 - > 20’187 | 10’439  7’874  >1’700 | [6, 7]  NC_019843  [4, 8-17] |
|  |  |  |  |  |  |  |
| Hepevirus |  |  |  |  |  |  |
|  | MT815970 | Bat hepevirus BtHEV-Ps1 strain/P.nathusii/Switzerland/2019 gene for nonstructural polyprotein, partial cds | Nonstructural polyprotein | < 1 – 4’667 | > 4’667 | [18]  NC_040835 |
|  |  |  |  |  |  |  |
| Parvovirus |  |  |  |  |  |  |
|  | MT815971 | Bat parvovirus strain BtNn-PV/Switzerland/2019 NS1 gene, partial cds | NS1 | <1 – 1’029 | <1’029 | [19]  NC_016744 |
|  | MT815972 | Bat parvovirus strain BtPk-PV/Switzerland/2019 NS1 and VP2 genes, complete cds | NS1  VP2 | 9 – 2’015  2’674 - 4’605 | 2’004  1’931 | [19]  NC_016744 |
|  | MT815973 | Bat parvovirus strain BtPn-PV/Switzerland/2019 NS1 gene, complete cds | NS1 | 98 – 2’086 | 1’988 | [19]  NC_016744 |
|  | MT815974 | Bat parvovirus strain BtPn-PV/Switzerland/2019 VP2 gene, partial cds | VP2 | <1 – 1’897 | 1’694 | [19]  NC_016744 |
|  | MT815975 | Bat parvovirus strain BtPp-PV/Switzerland/2019 NS1 gene, partial cds | NS1 | <1 - > 744 | > 744 | [19]  NC_016744 |
|  | MT815976 | Bat parvovirus strain BtPp-PV/Switzerland/2019 VP2 gene, partial cds | VP2 | <1 – 1’902 | 1’706 | [19]  NC_016744 |
|  | MT815977 | Bat parvovirus strain BtPa-PV/Switzerland/2019 NS1 gene, complete cds | NS1 | 186 – 2’192 | 2’006 | [19]  NC_016744 |
|  | MT815978 | Bat parvovirus strain BtPa-PV2/Switzerland/2019 NS1 gene, complete cds | NS1 | 60 – 2’066 | 2’006 | [19]  NC_016744 |
|  | MT815979 | Bat parvovirus strain BtPn-PV2/Switzerland/2019 VP2 gene, complete cds | VP2 | 674 – 2’599 | 1’925 | [19]  NC_016744 |
|  |  |  |  |  |  |  |
| Rotavirus A |  |  |  |  |  |  |
|  | MT815937 | Bat rotavirus strain BatRVA/Bat-wt/CH/Pip_kuh/2019 VP1 gene, partial cds | VP1 | <1 - >1’008 | > 1’008 | NC_040405 – 15  [20] |
|  | MT815938 | Bat rotavirus strain BatRVA/Bat-wt/CH/Rhi_fer/2019 VP1 gene, partial cds | VP1 | <1 - > 3’113 | > 3’113 | NC_040405 – 15  [20] |
|  | MT815939 | Bat rotavirus strain BatRVA/Bat-wt/CH/Rhi_hip/2019 VP1 gene, partial cds | VP1 | <1 - >1’938 | > 1’938 | NC_040405  [20] |
|  | MT815940 | Bat rotavirus strain BatRVA/Bat-wt/CH/Pip_pip/2019 VP1 gene, partial cds | VP1 | <1 - > 339 | > 339 | NC_040405  [20] |
|  | MT815941 | Bat rotavirus strain BatRVA/Bat-wt/CH/Pip_pip2/2019 VP2 gene, complete cds | VP2 | 15 – 2’663 | 2’648 | NC_040405  [20] |
|  | MT815942 | Bat rotavirus strain BatRVA/Bat-wt/CH/Rhi_fer/2019 VP2 gene, partial cds | VP2 | <1 - > 1’617 | > 1’617 | NC_040405  [20] |
|  | MT815943 | Bat rotavirus strain BatRVA/Bat-wt/CH/Rhi_hip/2019 VP2 gene, partial cds | VP2 | <1 - > 2’328 | > 2’328 | NC_040405  [20] |
|  | MT815944 | Bat rotavirus strain BatRVA/Bat-wt/CH/Rhi_fer/2019 VP3 gene, partial cds | VP3 | <1 - > 2’432 | >2’432 | NC_040405  [20] |
|  | MT815945 | Bat rotavirus strain BatRVA/Bat-wt/CH/Rhi_hip/2019 VP3 gene, partial cds | VP3 | <1- > 2’249 | >2’249 | NC_040405  [20] |
|  | MT815946 | Bat rotavirus strain BatRVA/Bat-wt/CH/Rhi_fer/2019 VP4 gene, partial cds | VP4 | <1 – 2’200 | >2’200 | NC_040405  [20] |
|  | MT815947 | Bat rotavirus strain BatRVA/Bat-wt/CH/Rhi_hip/2019 VP4 gene, partial cds | VP4 | <1 – 2’184 | >2’184 | NC_040405  [20] |
|  |  |  |  |  |  |  |
| Rotavirus H |  |  |  |  |  |  |
|  | MT815948 | Bat rotavirus strain BatRVH/Bat-wt/CH/Myo_dau/2019 NSP1 gene, complete cds | NSP1 | 22 - 1’224 | 1’202 | [21]  NC_007552 |
|  | MT815949 | Bat rotavirus strain BatRVH/Bat-wt/CH/Myo_dau2/2019 NSP1 gene, complete cds | NSP1 | 20 – 1’222 | 1’202 | [21]  NC_007552 |
|  | MT815950 | Bat rotavirus strain BatRVH/Bat-wt/CH/Myo_dau3/2019 NSP1 gene, complete cds | NSP1 | 34 – 1’236 | 1’202 | [21]  NC_007552 |
|  | MT815951 | Bat rotavirus strain BatRVH/Bat-wt/CH/Myo_dau/2019 NSP2 gene, complete cds | NSP2 | 24 - 914 | 890 | [21]  NC_007554 |
|  | MT815952 | Bat rotavirus strain BatRVH/Bat-wt/CH/Myo_dau2/2019 NSP2 gene, complete cds | NSP2 | 48 - 938 | 890 | [21]  NC_007554 |
|  | MT815953 | Bat rotavirus strain BatRVH/Bat-wt/CH/Myo_dau3/2019 NSP2 gene, complete cds | NSP2 | 73 – 963 | 890 | [21]  NC_007554 |
|  | MT815954 | Bat rotavirus strain BatRVH/Bat-wt/CH/Myo_dau/2019 NSP3 gene, complete cds | NSP3 | 83 - 799 | 716 | [21]  NC_007555 |
|  | MT815955 | Bat rotavirus strain BatRVH/Bat-wt/CH/Myo_dau2/2019 NSP3 gene, complete cds | NSP3 | 73 - 789 | 716 | [21]  NC_007555 |
|  | MT815956 | Bat rotavirus strain BatRVH/Bat-wt/CH/Myo_dau3/2019 NSP3 gene, complete cds | NSP3 | 33 – 833 | 800 | [21]  NC_007555 |
|  | MT815957 | Bat rotavirus strain BatRVH/Bat-wt/CH/Myo_dau3/2019 NSP4 gene, complete cds | NSP4 | 33 - 683 | 650 | [21]  NC_007557 |
|  | MT815958 | Bat rotavirus strain BatRVH/Bat-wt/CH/Myo_dau/2019 NSP5 gene, complete cds | NSP5 | 58 - 600 | 542 | [21]  NC_007558 |
|  | MT815959 | Bat rotavirus strain BatRVH/Bat-wt/CH/Myo_dau2/2019 NSP5 gene, complete cds | NSP5 | 55 - 597 | 542 | [21]  NC_007558 |
|  | MT815960 | Bat rotavirus strain BatRVH/Bat-wt/CH/Myo_dau3/2019 NSP5 gene, complete cds | NSP5 | 58 - 600 | 542 | [21]  NC_007558 |
|  | MT815961 | Bat rotavirus strain BatRVH/Bat-wt/CH/Myo_dau/2019 VP1 gene, complete cds | VP1 | 7 – 3’510 | 3’503 | [21]  NC_007548 |
|  | MT815962 | Bat rotavirus strain BatRVH/Bat-wt/CH/Myo_dau2/2019 VP1 gene, complete cds | VP1 | < 2 – 3’505 | > 3’503 | [21]  NC_007548 |
|  | MT815963 | Bat rotavirus strain BatRVH/Bat-wt/CH/Myo_dau3/2019 VP1 gene, complete cds | VP1 | 7 - > 2’710 | > 2’703 | [21]  NC_007548 |
|  | MT815964 | Bat rotavirus strain BatRVH/Bat-wt/CH/Myo_dau/2019 VP2 gene, complete cds | VP2 | 43 – 2’991 | 2’948 | [21]  NC_007549 |
|  | MT815965 | Bat rotavirus strain BatRVH/Bat-wt/CH/Myo_dau2/2019 VP2 gene, partial cds | VP2 | < 1 – 2’949 | > 2’949 | [21]  NC_007549 |
|  | MT815966 | Bat rotavirus strain BatRVH/Bat-wt/CH/Myo_dau/2019 VP3 gene, complete cds | VP3 | 9 – 2’168 | 2’159 | [21]  NC_007551 |
|  | MT815967 | Bat rotavirus strain BatRVH/Bat-wt/CH/Myo_dau3/2019 VP3 gene, complete cds | VP3 | 9 – 2’168 | 2’159 | [21]  NC_007551 |
|  | MT815968 | Bat rotavirus strain BatRVH/Bat-wt/CH/Myo_dau2/2019 VP4 gene, partial cds | VP4 | 197 – 2’449 | 2’252 | [21]  NC_007550 |
|  | MT815969 | Bat rotavirus strain BatRVH/Bat-wt/CH/Myo_dau/2019 VP6 gene, complete cds | VP6 | 33 – 1’233 | 1’200 | [21]  NC_007553 |

1. Sonntag M, Mühldorfer K, Speck S, Wibbelt G, Kurth A. New adenovirus in bats, Germany. Emerg Infect Dis. 2009;15(12):2052. doi: 10.3201/eid1512.090646.

2. Li Y, Ge X, Zhang H, Zhou P, Zhu Y, Zhang Y, et al. Host range, prevalence, and genetic diversity of adenoviruses in bats. J Virol. 2010;84(8):3889-97. doi: 10.1007/s11262-018-1577-9.

3. Hackenbrack N, Rogers MB, Ashley RE, Keel MK, Kubiski SV, Bryan JA, et al. Evolution and cryo-electron microscopy capsid structure of a North American bat adenovirus and its relationship to other mastadenoviruses. J Virol. 2017;91(2):e01504-16. doi: 10.1128/JVI.01504-16.

4. Wu Z, Yang L, Ren X, He G, Zhang J, Yang J, et al. Deciphering the bat virome catalog to better understand the ecological diversity of bat viruses and the bat origin of emerging infectious diseases. ISME J. 2016;10(3):609-20. Epub 2015/08/11. doi: 10.1038/ismej.2015.138.

5. He B, Li Z, Yang F, Zheng J, Feng Y, Guo H, et al. Virome profiling of bats from Myanmar by metagenomic analysis of tissue samples reveals more novel Mammalian viruses. PLoS One. 2013;8(4):e61950. Epub 2013/04/22. doi: 10.1371/journal.pone.0061950.

6. Zaki AM, Van Boheemen S, Bestebroer TM, Osterhaus AD, Fouchier RA. Isolation of a novel coronavirus from a man with pneumonia in Saudi Arabia. N Engl J Med. 2012;367(19):1814-20. doi: 10.1056/nejmx130029.

7. de Groot RJ, Baker SC, Baric RS, Brown CS, Drosten C, Enjuanes L, et al. Commentary: Middle east respiratory syndrome coronavirus (mers-cov): announcement of the coronavirus study group. J Virol. 2013;87(14):7790-2. doi: 10.1128/JVI.01244-13.

8. Yinda CK, Ghogomu SM, Conceição-Neto N, Beller L, Deboutte W, Vanhulle E, et al. Cameroonian fruit bats harbor divergent viruses, including rotavirus H, bastroviruses, and picobirnaviruses using an alternative genetic code. Virus Evol. 2018;4(1):vey008. Epub 2018/03/30. doi: 10.1093/ve/vey008.

9. Lau SK, Li KS, Tsang AK, Lam CS, Ahmed S, Chen H, et al. Genetic characterization of Betacoronavirus lineage C viruses in bats reveals marked sequence divergence in the spike protein of pipistrellus bat coronavirus HKU5 in Japanese pipistrelle: implications for the origin of the novel Middle East respiratory syndrome coronavirus. J Virol. 2013;87(15):8638-50. doi: 10.1128/JVI.01055-13.

10. Luo C-M, Wang N, Yang X-L, Liu H-Z, Zhang W, Li B, et al. Discovery of novel bat coronaviruses in South China that use the same receptor as Middle East respiratory syndrome coronavirus. J Virol. 2018;92(13): e00116-18. doi: 10.1128/jvi.00116-18.

11. Lau SK, Woo PC, Li KS, Huang Y, Wang M, Lam CS, et al. Complete genome sequence of bat coronavirus HKU2 from Chinese horseshoe bats revealed a much smaller spike gene with a different evolutionary lineage from the rest of the genome. Virol. 2007;367(2):428-39. doi: 10.1016/j.virol.2007.06.009.

12. Woo PC, Wang M, Lau SK, Xu H, Poon RW, Guo R, et al. Comparative analysis of twelve genomes of three novel group 2c and group 2d coronaviruses reveals unique group and subgroup features. J Virol. 2007;81(4):1574-85. doi: 10.1128/JVI.02182-06.

13. Chu D, Peiris J, Chen H, Guan Y, Poon LL. Genomic characterizations of bat coronaviruses (1A, 1B and HKU8) and evidence for co-infections in Miniopterus bats. J Gen Virol. 2008;89(5):1282-7. doi: 10.1099/vir.0.83605-0.

14. T ang X, Zhang J, Zhang S, Wang P, Fan X, Li L, et al. Prevalence and genetic diversity of coronaviruses in bats from China. J Virol. 2006;80(15):7481-90. doi: 10.1128/JVI.00697-06.

15. Obameso JO, Li H, Jia H, Han M, Zhu S, Huang C, et al. The persistent prevalence and evolution of cross-family recombinant coronavirus GCCDC1 among a bat population: a two-year follow-up. Science China Life Sciences. 2017;60(12):1357-63. doi: 10.1007/s11427-017-9263-6.

16. Tao Y, Shi M, Chommanard C, Queen K, Zhang J, Markotter W, et al. Surveillance of bat coronaviruses in Kenya identifies relatives of human coronaviruses NL63 and 229E and their recombination history. J Virol. 2017;91(5):e01953-16. doi: 10.1128/JVI.01953-16.

17. Drexler JF, Gloza-Rausch F, Glende J, Corman VM, Muth D, Goettsche M, et al. Genomic characterization of SARS-related coronavirus in European bats and classification of coronaviruses based on partial RNA-dependent RNA polymerase gene sequences. J Virol. 2010; 84(21): 11336-49. doi: 10.1128/JVI.00650-10.

18. Reuter G, Boros Á, Tóth Z, Kapusinszky B, Delwart E, Pankovics P. Detection of a novel RNA virus with hepatitis E virus-like non-structural genome organization in amphibian, agile frog (Rana dalmatina) tadpoles. Infect Genet Evol. 2018;65:112-6. doi: 10.1016/j.meegid.2018.07.029.

19. Canuti M, Eis-Huebinger AM, Deijs M, de Vries M, Drexler JF, Oppong SK, et al. Two novel parvoviruses in frugivorous New and Old World bats. PLoS One. 2011;6(12): e29140. doi: 10.1371/journal.pone.0029140.

20. Small C, Barro M, Brown TL, Patton JT. Genome heterogeneity of SA11 rotavirus due to reassortment with “O” agent. Virol. 2007;359(2):415-24. doi: 10.1016/j.virol.2006.09.024.

21. Jiang S, Ji S, Tang Q, Cui X, Yang H, Kan B, et al. Molecular characterization of a novel adult diarrhoea rotavirus strain J19 isolated in China and its significance for the evolution and origin of group B rotaviruses. J Gen Virol. 2008;89(10):2622-9. doi: 10.1099/vir.0.2008/001933-0.
